# VCCV: conservative transcriptomic corroboration for measurement prioritization of computational drug–target hypotheses

**DOI:** 10.1093/bioinformatics/btag694

**Published:** 2026-09-18

**Authors:** Haihui Huang, Yanan Zhou, Dingkui Kang, Yong Liang

**Affiliations:** Shaoguan Research Center of the National Engineering Laboratory for Big Data System Computing Technology, Shaoguan University, Shaoguan, 512005, China; Guangdong Provincial Laboratory of Traditional Chinese Medicine, Hengqin, 519031, China; Shaoguan Research Center of the National Engineering Laboratory for Big Data System Computing Technology, Shaoguan University, Shaoguan, 512005, China; Guangdong Provincial Laboratory of Traditional Chinese Medicine, Hengqin, 519031, China; Guangdong Provincial Laboratory of Traditional Chinese Medicine, Hengqin, 519031, China; Guangdong Provincial Laboratory of Traditional Chinese Medicine, Hengqin, 519031, China; College of Mechatronics and Control Engineering, Shenzhen University, Shenzhen, 518061, China

## Abstract

**Motivation:**

Computational drug-target interaction (DTI) models nominate plausible binders but cannot determine which candidate best accounts for an observed cellular response. Perturbational transcriptomics offers orthogonal mechanistic evidence, yet pharmacology-to-genetics mismatch, incomplete reference coverage, and non-specific stress programs make simple signature matching unreliable. This motivates a principled integration layer that corroborates hypotheses conservatively, abstains under global mismatch, and prioritizes informative follow-up measurements when the evidence remains ambiguous.

**Results:**

We present Virtual-to-Cellular Corroboration for Validation (VCCV), a model-agnostic posterior-triage layer for pre-trained DTI models. VCCV updates calibrated DTI working weights with context-aligned perturbational evidence using a near-identity affine map and exact covariance transport. Within the stated Gaussian class, exact transport is the unique uncertainty update that preserves posterior-odds comparisons under invertible affine changes of measurement coordinates. VCCV also introduces an empirical warning branch for abstention and converts residual ambiguity into compact follow-up gene panels, using a submodular objective for deep near-ties. Each query is assigned one of three actionable states: a target-resolved hypothesis, an abstention, or a prioritized panel. Across five DTI models, VCCV improved discrimination (paired ROC-AUC gains 0.021–0.037) and reduced negative log-likelihood in every case. Additional evaluations showed improved ranking on the same-cell-supported endpoint, warning-score discrimination of strong-response profiles (ROC-AUC 0.876), and better recovery of full-coordinate leading hypotheses by selected panels than by random panels. Across these retrospective kinase-focused evaluations, VCCV provides a principled bridge from computational nomination to conservative, measurement-directed cellular corroboration.

**Availability and implementation:**

Source code of VCCV is publicly available at https://github.com/bio-ai-source/VCCV.

## 1 Introduction

Identifying the precise molecular targets through which a bioactive compound exerts its phenotypic effects remains a central challenge in chemical biology and early-stage drug discovery, particularly when computational drug–target interaction (DTI) and binding-prediction models nominate candidates before cellular mechanisms have been experimentally resolved (Swinney and Anthony 2011, Schenone *et al.* 2013, Anighoro *et al.* 2014, Kubota *et al.* 2019, Ouyang *et al.* 2024, Liu *et al.* 2025, Yu Q *et al.* 2025). Because such nominations reflect biophysical compatibility rather than confirmed cellular mechanisms, target deconvolution is better cast as a staged decision problem under uncertainty: a DTI model supplies calibrated working weights, and cellular assays must update them conservatively. A robust framework should strengthen a nomination only when orthogonal evidence supports it, withhold support when the response is globally mismatched or stress-associated, and indicate what to measure next when the mechanism remains ambiguous.

Deep learning has substantially improved computational DTI prediction. Architectures ranging from sequence- and graph-based encoders (Huang *et al.* 2021, Nguyen *et al.* 2021) to unified and evidential frameworks (Lu *et al.* 2025, Shah *et al.* 2025, Zhao *et al.* 2025) excel at predicting drug–target interactions and binding affinities, and related studies have extended graph-based modelling to drug-interaction and cellular drug-response prediction (Le 2023, Tran *et al.* 2025). DTI affinity predictions nonetheless remain context-blind: two compounds with identical predicted affinity profiles may elicit divergent cellular consequences depending on lineage, exposure duration, dose-dependent feedback, and uncharacterized off-target liabilities. DTI models therefore serve as hypothesis generators whose predictions remain incomplete without empirical cellular corroboration.

Perturbational transcriptomics, exemplified by the Connectivity Map (Lamb *et al.* 2006) and LINCS L1000 (Subramanian *et al.* 2017, Stathias *et al.* 2020), captures the global cellular state following an intervention. In principle, matching a compound’s transcriptomic footprint to the reference signature of a genetic perturbation of its putative target provides mechanistic corroboration. In practice, however, pharmacological and genetic interventions differ in onset kinetics, penetrance, and pleiotropy, and high-dose treatments trigger non-specific stress responses that can overwhelm target-specific signals; naive signature matching therefore conflates shared stress-associated programs with genuine mechanistic concordance. Transcriptomics-aware frameworks such as FRoGS (Chen *et al.* 2024) and GMPTI (Li *et al.* 2022) show that gene-signature information is informative for target nomination, while PRnet (Qi *et al.* 2024) and PerturbNet (Yu H *et al.* 2025) illustrate broader progress in learned perturbation-response modelling. Transcriptomic foundation models such as Geneformer (Theodoris *et al.* 2023) and scGPT (Cui *et al.* 2024) supply increasingly general cell-state representations, but none, in its published form, jointly evaluates competing calibrated hypotheses on identical DTI candidate rows while also providing an explicit null branch and a measurement-selection state. Numerical comparisons here are therefore restricted to controls evaluable on the same candidate rows. To our knowledge, no existing method jointly provides a formal posterior update from calibrated DTI working weights, an explicit abstention mechanism for global lack of fit, and an ambiguity-conditioned policy for follow-up measurement.

The core methodological challenge is to identify the formal properties such an integration must satisfy to remain conservative. Transcriptomic measurements carry baseline shifts and scaling artifacts across platforms: transformations equivalent to invertible affine coordinate changes. We establish (Theorem 1) that if posterior odds between mechanistic hypotheses are to remain invariant under such coordinate changes, uncertainty must be transported by exact covariance push-forward $\left(\Sigma \mapsto A\Sigma A^{⊤}\right)$; a marginal-diagonal approximation cannot satisfy this invariance universally. For a fixed branch mean under the exact Gaussian branch model, approximating this covariance incurs a nonnegative model-expected log-score penalty equal to a Kullback–Leibler divergence; under strict diagonalization, the penalty reduces to $-1/2\mathrm{logdet}C_{h}$, where $C_{h}$ is the branch correlation matrix.

Three methodological consequences follow. First, the fixed-mass rule shifts every retained target-versus-nontarget log posterior odds by exactly $-\log S_{K}(x)$ relative to its matched absolute-weight reference, where $S_{K}(x)$ is the retained target-weight sum; this shift is target-favouring if and only if $S_{K}(x)<1$ (sharpened Corollary 1). Second, within a posterior normalized only over target branches and lacking any external absolute-fit or rejection channel, a common shift of all target energies is invisible, so an external rejection channel is required; this motivates the explicit empirical warning/null branch described below (Corollary 2). Third, deleting the null or polypharmacology branch, or rescaling all retained target weights by a common query-specific factor, leaves every within-query single-target ranking metric exactly unchanged; the corresponding within-query ranking contrasts must therefore be zero, even though these changes can still affect pooled discrimination, calibration, target-versus-nontarget competition, posterior gaps, and returned decision states.

Guided by these constraints, we introduce Virtual-to-Cellular Corroboration for Validation (VCCV), a conservative measurement-prioritization layer applicable to any pre-trained DTI predictor. Across three decision states (Fig. 1), VCCV retains a target-resolved branch when transcriptomic support is sufficiently separated under the fitted model, shifts posterior mass to a weighted empirical warning branch under global mismatch, and formulates residual near-ties as a submodular minimum-falsification objective that converts unresolved ambiguity into compact gene panels for follow-up. Across five DTI models and a filtered same-cell-supported linked endpoint, VCCV refines target discrimination, supports abstention in warning-compatible responses, and translates computational uncertainty into prioritized measurement strategies. Tables S1–S5 report sensitivity analyses and calibration settings, S6 linked-endpoint uncertainty, S7 warning-score sensitivities, S8 and S8A linked-endpoint ranking, S9 model inputs, and S10–S18 paired effects, scope assessments, panels, parameters, ablations, and map diagnostics.

**Figure 1:**
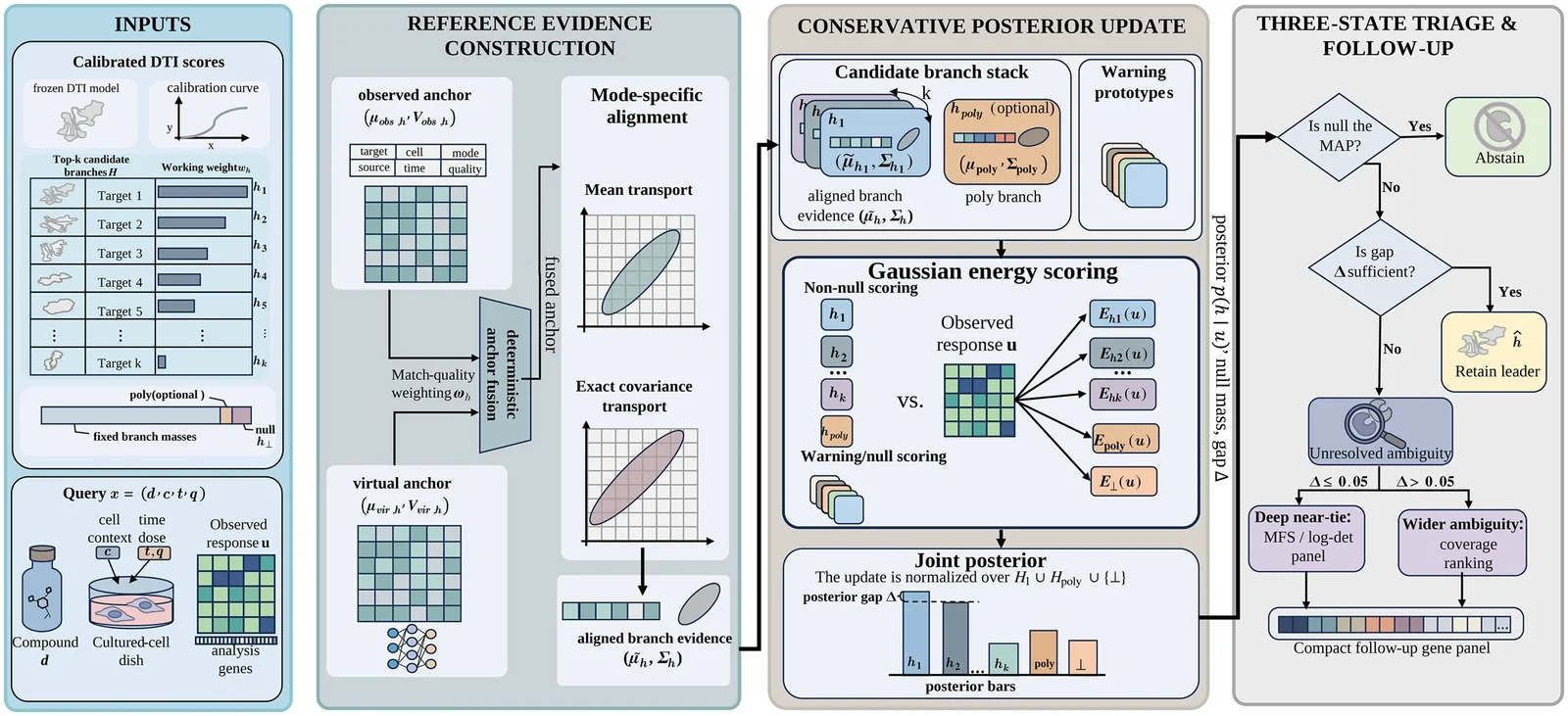
Decision logic of VCCV. A calibrated DTI score is evaluated against context-aligned perturbation evidence. Joint posterior triage returns a target-resolved state, a warning/abstention state, or an ambiguity-conditioned follow-up panel.

## 2 System and methods

### 2.1 Query, hypothesis space, and decision rule

For a biological query x=(d,c,t,q), d denotes the compound, c the cellular context, t the exposure time, and q the compound dose in micromolar units; u is the measured transcriptomic response vector across G analysis coordinates. Each pre-trained DTI model is mapped to calibration-split probabilities by model-specific Platt scaling (Platt 2000), and, when multiple calibrated DTI channels are available, a calibration-only selector chooses among a fixed family of fusion rules. For each query, the top K targets are retained. Let $H_{1}(x)$ denote the candidate space of retained single-target mechanisms and $w_{h}(x)>0$ the calibrated working weight of branch h. Conditioning on x is suppressed below, so $p_{\eta }(h|u)$ denotes $p_{\eta }(h|u,x)$. VCCV uses the calibrated scores without updating the underlying DTI model; model input modalities are summarized in Table S9.

Retained target weights are normalized within a prespecified single-target mass $m_{1}=0.8$, so that $\pi_{h}^{\mathrm{fix}}(x)=0.8w_{h}(x)/S_{K}(x)$, while the optional polypharmacology branch and the warning/null branch each receive fixed mass 0.1. Relative to the matched absolute-weight rule $\pi_{h}^{\mathrm{abs}}(x)=0.8w_{h}(x)$, sharpened Corollary 1 gives the exact shift of every retained target-versus-nontarget log posterior odds as $-\log S_{K}(x)$, which is target-favouring if and only if $S_{K}(x)<1$. Since $1/S_{K}(x)$ is common to all retained targets, target-to-target odds are unchanged. The fixed-mass rule is used in the main analyses; the absolute-weight alternative is evaluated on the same candidate rows and with the same calibration procedure (Supplementary H.9).

The VCCV formulation can additionally retain an optional polypharmacology branch $H_{\mathrm{poly}}$ when a weighted multi-target explanation remains competitive, and it always includes an explicit null branch ⊥ for stress-dominated or otherwise non-target-resolved responses. The full decision space is $H=H_{1}\cup H_{\mathrm{poly}}\cup \{⊥\}$, and joint posterior normalization runs over all three parts. Support for one branch consequently reduces support for the others, and every posterior statement is explicitly comparative. Each pre-trained DTI model is evaluated separately in the core analyses.

### 2.2 Observed and virtual perturbation evidence

For each single-target branch, the perturbational layer provides an observed anchor when a supported LINCS genetic perturbation is available, a virtual anchor otherwise, or both when empirical support is partial. The virtual model is trained on quality-controlled LINCS short hairpin RNA knockdown and gene overexpression signatures aggregated by target, cell context, perturbation mode, and exposure time within the training split. It is a conditional heteroscedastic regressor that predicts an expected response and gene-wise uncertainty from target, context, mode, and time. Compound dose is not used as a genetic-anchor covariate because genetic delivery amounts are recorded in non-comparable units. Parameters are estimated by gene-wise Gaussian negative log-likelihood with a standard-deviation floor, and a single virtual-variance factor is fixed using the calibration split. Target/context combinations lacking both observed and virtual support are omitted.

Observed and virtual anchors are fused by a deterministic match-quality rule. Candidate target signatures must match the query cell line; records from the same source are prioritized, and the nearest exposure time is then selected, with platform, batch, and replicate quality used to quantify support. Drug concentration is never compared with genetic-delivery volume or mass. We write the fused moments as $\left(\mu_{\mathrm{fuse},h},v_{\mathrm{fuse},h}\right)$ and use them in the alignment step; the moment-fusion rule and anchor-quality definition are given in Supplementary A.3.

To account for systematic offsets between the pharmacological and genetic perturbation spaces, VCCV maps the fused reference into the compound-response space via a mode-specific near-identity affine map. Systematic pharmacology–genetics offsets are modelled by one shared near-identity matrix $B=I+UV^{⊤}$ on the prespecified G=256 measured-landmark representation, with mode-specific scale $\beta_{m}$ and bias $b_{m}$; the fitted update B-I has numerical rank 4. The aligned mean is ${\tilde{\mu }}_{h}=\beta_{m}B\mu_{\mathrm{fuse},h}+\beta_{m}b_{m}$. For a single-target branch with fused variance $v_{\mathrm{fuse},h}$, the scored covariance is the exact push-forward:


(1) $$\Sigma_{h}=s[\beta_{m}^{2}B\text{diag}(v_{\mathrm{fuse},h})B^{⊤}+\Sigma_{\mathrm{base}}],$$

where $\Sigma_{\mathrm{base}}≻0$ is a fixed base noise covariance and the covariance scale s=0.033 is estimated for Section 4.1 from drug-held-out training residuals and fixed before calibration and testing. The polypharmacology covariance is obtained by Gaussian moment matching of its constituent single-target moments, including between-mean dispersion. Single-target branches thus retain the structured covariance induced by alignment, consisting of a positive-definite base term plus an augmented low-rank update of latent dimension at most 2r=8; quadratic forms and log determinants are evaluated exactly through the Woodbury and Sylvester reductions of Supplementary B.1. Since $∥B-I{∥}_{2}=0.761<1$, the map used in the Section 4.1 analysis satisfies the admissibility condition of Proposition H1 and requires no spectral projection. The separately fitted target-disjoint map in Supplementary H.9 is constrained at $∥B-I{∥}_{2}\leq 0.95$ and undergoes 55 projections during estimation (Section 4.3; Supplementary H.9–H.10). The inverse temperature η=0.05 and positive-slope calibration are estimated for Section 4.1 using only the relevant calibration partition; Table S3 applies to the analysis reported in Section 4.1. Theorem 1 shows that this exact covariance transport is the unique branchwise update preserving posterior-odds comparisons under admissible changes of response coordinates.

### 2.3 Conservative posterior, explicit null, and decision states

Each non-null branch h is scored by a Gaussian energy:


(2) $$E_{h}(u)=\frac{1}{2}{\left(u-{\tilde{\mu }}_{h}\right)}^{⊤}\Sigma_{h}^{-1}(u-{\tilde{\mu }}_{h})+\frac{1}{2}\log\;\det\;\Sigma_{h}.$$

The warning/null branch aggregates a training-only library of empirical prototypes with fixed cluster-frequency weights $w_{k}$:


(3) $$E_{⊥}(u)=-\log\sum_{k}w_{k}e\mathrm{xp}\;\{-E_{⊥k}(u)\}.$$

This training-derived weighted soft-min supports abstention when represented target branches fit poorly or the response is warning-compatible. Scoring is determined by the prototype set and its cluster-frequency weights; post hoc prototype annotations remain descriptive.

The conservative posterior is $p_{\eta }(h|u)=\pi (h)e\mathrm{xp}[-\eta E_{h}(u)]/Z(u)$ for h∈H with h≠⊥, and $p_{\eta }(⊥|u)=\pi (⊥)e\mathrm{xp}[-\eta E_{⊥}(u)]/Z(u)$, with joint normalization over all branches in H. The inverse temperature η>0 controls the strength of the transcriptomic update to the calibrated DTI working weights.

### 2.4 Ambiguity-conditioned follow-up panels

Let $\hat{h}=\arg\max_{h\in H}p_{\eta }(h|u)$ denote the global maximum a posteriori (MAP) branch. If $\hat{h}=⊥$, VCCV returns the warning/abstention state. For a non-null leader, define the posterior gap against all remaining branches, explicitly including the null, as $\Delta =p_{\eta }(\hat{h}|u)-\max_{h\neq \hat{h}}p_{\eta }(h|u)$. The leader is retained when $\Delta \geq \rho_{\mathrm{dec}}=0.42$; otherwise the query is unresolved. Retention is governed by this single gap rule: the keep-probability threshold $\gamma_{\mathrm{keep}}$ is disabled, and a null-MAP case with $p_{\eta }(⊥|u)\geq \gamma_{\mathrm{null}}=0.95$ is labelled strong-warning for descriptive reporting only.

For an unresolved query, the ambiguity set uses the same calibrated gap as the retention rule: $A_{\Delta }(x)=\{h\in H:p_{\eta }(\hat{h}|u)-p_{\eta }(h|u)\leq \rho_{\mathrm{dec}}\}$. Each member is represented by one Gaussian moment pair before panel construction: the aligned single-target pair, the moment-matched polypharmacology pair, or the highest-responsibility warning component $k^{*}=\arg\max_{k}\{\log w_{k}-E_{⊥k}(u)\}$. The panel stage therefore operates on a fully moment-specified ambiguity set.

Let $p_{h}=p_{\eta }(h|u)/\sum_{h\prime \in A_{\Delta }(x)}p_{\eta }(h\prime |u)$. For each gene g, define ${\overset{↼}{\mu }}_{g}=\sum_{h}p_{h}\mu_{h,g}$, $b_{g}=\sum_{h}p_{h}{\left(\mu_{h,g}-{\overset{↼}{\mu }}_{g}\right)}^{2}$, and $w_{g}=\sum_{h}p_{h}{\left(\Sigma_{h}\right)}_{gg}$. The standardized separation score is $s_{g}=b_{g}/(w_{g}+\varepsilon_{w})$, D=diag(s), and $R_{\mathrm{psd}}$ is the nearest positive-semidefinite projection of the empirical global gene-correlation matrix. Candidate genes are screened separately so that their pairwise correlation with every already selected gene remains below the collinearity ceiling $\rho_{\mathrm{panel}}=0.95$. Here $\varepsilon_{w}>0$ is used as a numerical stabilizer, while $\lambda_{\mathrm{panel}}\geq 0$ and $\lambda_{\mathrm{cov}}\geq 0$; the former provides the log-determinant ridge, and the latter weights the scale-free coverage penalty. The panel kernel is $L=D^{1/2}R_{\mathrm{psd}}D^{1/2}+\lambda_{\mathrm{panel}}I$, and for |S|≤k the Minimum Falsification Set (MFS) objective is $J(S)=\mathrm{logdet}(I+L_{S})$. The submodularity and 1-1/e guarantee apply to the cardinality-only log-determinant objective; the additional pairwise-correlation screen used during panel selection is a heuristic feasibility rule and is not covered by that guarantee.

The panel policy uses this separation-focused MFS objective only in the deep near-tie regime (Δ≤0.05). For wider ambiguity cases, it switches to a scale-free coverage-regularized variance order, ranking candidate genes by


$$U(g|S)={\tilde{v}}_{g}-\frac{\lambda_{\mathrm{cov}}}{\max\;\left\{1,\;|S|\right\}}\sum_{s\in S}|R_{g,s}|,$$

where ${\tilde{v}}_{g}\in [0,1]$ is the min–max normalized marginal posterior variance of gene g and $R_{g,s}$ its Pearson correlation with genes already selected. The penalty term is zero when S is empty. The VCCV algorithm is summarized as follows:

Algorithm 1 VCCV posterior triage and ambiguity-conditioned measurement prioritization.

**Input:** query response u, metadata x=(d,c,t,q), calibrated DTI working weights over $H_{1}$, fixed target/poly/null branch masses 0.8/0.1/0.1, observed and virtual anchors, alignment parameters, warning prototypes, and optional panel budget k.

**Output:** posterior $p_{\eta }(\cdot |u)$ over $H=H_{1}\cup H_{\mathrm{poly}}\cup \{⊥\}$, decision state in {retain, abstain, panel}, and optional panel S.

**Candidate construction.** Build the calibrated top-K DTI candidate set $H_{1}$ and append the optional $H_{\mathrm{poly}}$ and the mandatory ⊥.

**Target-branch scoring.** Retrieve the best observed anchor, predict a supported virtual anchor, and compute the deterministic observed weight $\omega_{h}$ for each single-target branch. Fuse anchors to obtain $\mu_{\mathrm{fuse},h}$ and $v_{\mathrm{fuse},h}$, then transport both mean and uncertainty through the near-identity alignment map to obtain ${\tilde{\mu }}_{h}$ and $\Sigma_{h}$ by Equation (1). Moment-match the optional polypharmacology branch, then evaluate $E_{h}(u)$ by Equation (2).

**Null scoring and posterior normalization.** Evaluate the cluster-frequency-weighted warning mixture by Equation (3), then normalize all retained target, polypharmacology, and warning branches jointly.

**Decision.** Compute $\hat{h}=\arg\max_{h\in H}p_{\eta }(h|u)$; if $\hat{h}=⊥$, abstain. Compute the posterior gap $\Delta =p_{\eta }(\hat{h}|u)-\max_{h\neq \hat{h}}p_{\eta }(h|u)$ against all competing branches. If $\Delta \geq \rho_{\mathrm{dec}}$, retain $\hat{h}$; otherwise build the gap-defined moment-specified ambiguity set $A_{\Delta }(x)$. For deep near-ties (Δ≤0.05), greedily maximize $J(S)=\mathrm{logdet}(I+L_{S})$ to obtain an MFS; otherwise rank genes by U(g∣S).

### 2.5 Algorithm and theory

Proposition 1 decomposes posterior odds into prior odds and transcriptomic energy differences. Theorem 1 establishes that exact covariance push-forward is the unique Gaussian uncertainty transport under affine representation invariance, with the invertibility and conditioning bound for the near-identity map given in Supplementary Proposition H1. Sharpened Corollary 1 gives the exact target-versus-nontarget odds shift induced by matched target-weight normalization, while Corollary 2 formalizes why an external rejection channel is needed; VCCV implements that channel with an explicit warning/null branch. Proposition 2 gives the cardinality-constrained log-det submodularity guarantee (Nemhauser *et al.* 1978). Proposition 3 identifies changes that leave within-query rankings unchanged, and Proposition 4 separates pointwise covariance-approximation distortion from its nonnegative model-expected proper-score penalty.

Proposition 1. *Posterior-odds decomposition.* For any two branches h and h′ with positive prior mass, $\log\frac{p_{\eta }(h|u)}{p_{\eta }(h\prime |u)}=\log\frac{\pi (h)}{\pi (h\prime )}-\eta [E_{h}(u)-E_{h\prime }(u)]$. In particular, h exceeds the null by a log-odds margin δ if and only if $E_{⊥}(u)-E_{h}(u)\geq \eta^{-1}(\log[\pi (⊥)/\pi (h)]+\delta )$.

Theorem 1. *Representation-invariant conservative corroboration.* Let u′=Au+b with A invertible. Transform each non-null Gaussian branch by $\mu_{h}\prime =A\mu_{h}+b$ and $\Sigma_{h}\prime =A\Sigma_{h}A^{⊤}$, and each null prototype by $\nu_{j}\prime =A\nu_{j}+b$ and $\Omega_{j}\prime =A\Omega_{j}A^{⊤}$. Then every posterior odds ratio between any two branches in H is unchanged. Conversely, within the positive-definite covariance-only class formalized in Supplementary B.3 (with the affine mean map, priors, and η fixed), preserving these odds for all invertible A uniquely forces the exact push-forward $\Sigma \mapsto A\Sigma A^{⊤}$.

Corollary 1. *Exact effect of matched target-weight normalization.* Let $w_{h}(x)>0$, let $S_{K}(x)$ be their retained sum over $h\in H_{1}(x)$, and let $m_{1}>0$. Compare $\pi_{h}^{\mathrm{fix}}(x)=m_{1}w_{h}(x)/S_{K}(x)$ with $\pi_{h}^{\mathrm{abs}}(x)=m_{1}w_{h}(x)$, holding all non-target branch weights and all branch energies fixed. For any retained target h and unchanged non-target branch b, the fixed rule shifts the log posterior odds by exactly $-\log S_{K}(x)$; equivalently, the minimum energy advantage $E_{b}(u)-E_{h}(u)$ required for any fixed log-odds margin changes by $\eta^{-1}l\mathrm{og}S_{K}(x)$. The rule is target-favouring if and only if $S_{K}(x)<1$, neutral at $S_{K}(x)=1$, and target-disfavouring if and only if $S_{K}(x)>1$, with the target-favouring shift diverging as $S_{K}(x)$ approaches zero from above.

Corollary 2. A posterior normalized only over target branches is invariant to adding the same constant to every target energy. Without an external absolute-fit or rejection channel, it therefore cannot convert common deterioration into abstention; VCCV supplies that channel through the explicit null branch.

Proposition 2. If L is positive semidefinite, then $J(S)=\mathrm{logdet}(I+L_{S})$ is monotone and submodular, and under |S|≤k greedy selection attains the standard 1-1/e approximation bound (Nemhauser *et al.* 1978). This approximation guarantee does not extend to the additional pairwise-correlation screen used by the practical panel policy.

Proposition 3. *Conditional invariance of within-query single-target ranking.* Fix a biological query (u,x), its retained set $H_{1}(x)$, the target energies, and positive target working weights. Any variant that changes only branches outside $H_{1}(x)$, or multiplies every retained target weight by the same positive query-specific factor, preserves $p_{\eta }(h|u,x)/p_{\eta }(g|u,x)$ for all $h,g\in H_{1}(x)$. Consequently every rank-only statistic on the same single-target rows, including within-query ROC-AUC, average precision, reciprocal rank, and top-k order, is identical, and any strictly increasing scalar calibration applied identically to those rows preserves this. The result does not extend to global pooled discrimination, calibration, binary-label negative log-likelihood (NLL), posterior gaps, target-versus-null/poly competition, or returned decision states.

Proposition 4. *Covariance-approximation distortion.* Let $\Sigma_{h}$ and ${\tilde{\Sigma }}_{h}$ be the exact and approximating positive-definite covariances for a branch with unchanged mean, and let $\delta_{h}(u)$ be the induced energy difference. Then $\delta_{h}(u)$ is query dependent and can have either sign, whereas under the exact branch model $E[\delta_{h}(U)|h]=D_{\mathrm{KL}}[N(\mu_{h},\Sigma_{h})\|N(\mu_{h},{\tilde{\Sigma }}_{h})]\geq 0$, with equality if and only if ${\tilde{\Sigma }}_{h}=\Sigma_{h}$. For ${\tilde{\Sigma }}_{h}=\mathrm{diag}(\Sigma_{h})$, the penalty reduces to $-1/2\mathrm{logdet}C_{h}\geq 0$ by Hadamard’s inequality, where $C_{h}$ is the branch correlation matrix. The query-dependent quadratic distortion cannot in general be removed by fixed prior shifts or a single global temperature. The statement is a model-conditional proper-score result and does not imply pointwise or ROC-AUC dominance.

Full derivations and proofs appear in Supplementary Sections A–C and H.

## 3 Data resources, split protocol, and evaluation design

Five retrospective estimands are evaluated separately: (i) proxy discrimination across pre-trained DTI models; (ii) linked same-cell-supported strong-versus-weak affinity ranking; (iii) real chemical-perturbation and external warning-score compatibility; (iv) target-disjoint component attribution; and (v) masked-coordinate panel prioritization. The warning, component, and panel analyses use the model described in Supplementary H.9, while each retains its own estimand and sampling unit. Davis and KIBA supply biochemical labels, whereas LINCS L1000 supplies perturbational evidence (Davis *et al.* 2011, Tang *et al.* 2014, Subramanian *et al.* 2017, Stathias *et al.* 2020). Gene-rank cosine similarity and the weighted target-up/down rank score are evaluated on the same linked candidate space. These endpoints evaluate transcriptomic corroboration and measurement prioritization, with the evidentiary scope of each analysis detailed in Supplementary Section F.

The DTI-score control is the calibrated DTI-model score evaluated directly on the linked candidate space without transcriptomic updating. The context-aware fusion control uses the same linked candidate space and perturbational inputs while omitting the full null-aware VCCV triage rule. For gene-rank cosine similarity and weighted target-up/down rank score, a monotone sign correction is applied on the calibration split so that larger scores consistently favor the positive class before computing discrimination metrics. The signature-score and fusion baselines include neither a null branch nor a panel state and therefore act as candidate-ranking comparators; their score mappings, missing-score handling, and the class-balanced fusion model are specified in Supplementary H.3.

Label-independent profile transforms precede splitting, after which the relevant entity or grouping split is fixed. Scaling, alignment, fitted-reference, covariance, and warning quantities are estimated only on their designated fitting partitions and applied without refitting; calibration-derived quantities are selected only on the calibration partition and fixed before test scoring; endpoint-specific panel constants, masks, and eligibility rules are fixed before the held-out-coordinate evaluation. Split consistency is verified by confirming zero overlap for the named held-out unit in each regime. Because Davis and KIBA contain no measured cell, time, or dose, the deterministic context-like and condition-like labels are sensitivity groupings rather than biological hold-outs. The assessment uses calibrated scores supplied by the published DTI models and does not assess their original training data. The linked target-row diagnostic is transductive, whereas the analysis in Supplementary H.9 uses target-disjoint map fitting with fixed evaluation-time target references (Supplementary H.4).

## 4 Results

### 4.1 Conservative corroboration improves discrimination across pre-trained DTI models

Table 1 reports the absolute pooled ROC-AUC and pooled binary NLL (pNLL) of each pre-trained DTI model and its VCCV update. Paired ΔROC-AUC estimates and 95% confidence intervals are given in the text and Table S10; Fig. 2a shows paired pNLL reduction, and Fig. 2b summarizes gains across grouping regimes. ROC-AUC gains are 0.021–0.037 and pNLL is reduced for every DTI model; mean ΔROC-AUC is positive across drug hold-out (+0.039), deterministic context-like grouping (+0.020), target partition (+0.019), and deterministic condition-like shift (+0.017). The context-like and condition-like labels are sensitivity groupings and do not represent measured Davis/KIBA conditions.

**Figure 2:**
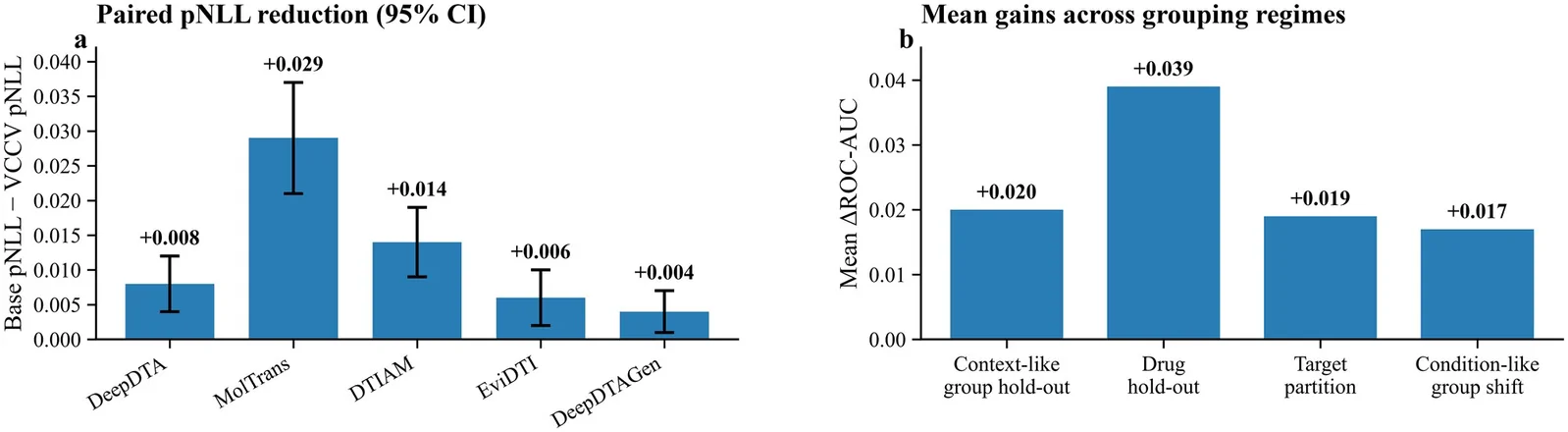
Conservative corroboration across pre-trained DTI models. Panel a: paired pooled binary negative log-likelihood (pNLL) reduction with 95% confidence intervals. Panel b: mean ROC-AUC gain across entity or deterministic grouping regimes; context-like and condition-like labels denote sensitivity groupings, not measured Davis/KIBA biology. Exact paired effects are reported in Table S10; Table S1 defines the grouping regimes and their interpretive scope.

**Table 1:** Transcriptomic corroboration across DTI models.

Model	ROC-AUC	pNLL
DeepDTA (Öztürk *et al.* 2018)	0.811	0.105
VCCV + DeepDTA	0.834	0.097
MolTrans (Huang *et al.* 2021)	0.825	0.106
VCCV + MolTrans	0.862	0.077
DTIAM (Lu *et al.* 2025)	0.841	0.085
VCCV + DTIAM	0.868	0.071
EviDTI (Zhao *et al.* 2025)	0.844	0.080
VCCV + EviDTI	0.875	0.074
DeepDTAGen (Shah *et al.* 2025)	0.854	0.090
VCCV + DeepDTAGen	0.875	0.086

*Note.* Paired VCCV effects with 95% confidence intervals are reported in Table S10.

After resolving 489 duplicate canonical pairs, nine conflicting labels, and one identifier collision, continuous affinities were aggregated by canonical drug-target pair before one binary thresholding step. Davis contains 12 000 pairs (1037 positive; 8.642%), whereas KIBA contains 12 000 pairs (11 685 positive; 97.375%); their class imbalance is therefore reported separately. The paired analysis uses a common set of 81 185 deduplicated rows representing 22 707 dataset instances and 1653 drug clusters; uncertainty is estimated with 2000 paired drug-cluster bootstrap resamples stratified by scenario × seed × dataset. The paired ΔROC-AUC estimates are +0.023 (95% CI 0.015–0.031) for DeepDTA, +0.037 (0.026–0.048) for MolTrans, +0.027 (0.018–0.036) for DTIAM, +0.031 (0.020–0.042) for EviDTI, and +0.021 (0.011–0.031) for DeepDTAGen; all five intervals exclude zero, and paired pNLL reductions are positive (Table S10). Because the gains recur across five architecturally distinct DTI models and every evaluated grouping regime, the results support a contribution from the transcriptomic update that is consistent across the evaluated DTI models.

### 4.2 Warning-score compatibility with strong or stress-associated responses

The final warning-score evaluation uses only real LINCS chemical perturbations recorded in micromolar units and the exact-covariance model specified in Supplementary H.9, with η=0.003 fixed from the calibration partition before test evaluation; genetic-delivery quantities are excluded from matching. Among 305 chemical signatures representing 39 drugs, the strong-response label was defined as a LINCS signature-strength count greater than 6 (more than six significantly differentially expressed transcripts), without using warning probabilities (eight positives). Because the label is derived from the same expression profiles, this analysis provides a convergent response-strength assessment based on metadata defined without using warning probabilities. The warning probability achieved ROC-AUC 0.876 (drug-cluster bootstrap 95% CI 0.778–1.000) and average precision (AP) 0.258 (Table 2; Supplementary E.7). For the transcriptional activity score (TAS) ≥0.2 endpoint, the warning score yielded ROC-AUC 0.552 (95% CI 0.443–0.660; Table S7).

**Table 2:** Real-compound and external warning-score analyses.

Evaluation	Estimate	Interpretation
Real LINCS chemical responses	ROC-AUC 0.876 [0.778, 1.000]; AP 0.258	Same-profile convergent response-strength assessment using a warning-independent LINCS signature-strength threshold (>6 significantly differentially expressed transcripts); 10 000 drug-cluster bootstrap.
External Tox21 common condition	ROC-AUC 0.728 [0.573, 0.862]	Metadata-selected common-condition stress-activity assessment
External ToxCast common condition	Spearman ρ = 0.727 [0.229, 0.956]; *P* = 0.011; binary sensitivity AUC 0.719	Continuous association at the metadata-selected common condition; no dose-aware cytotoxicity endpoint

*Note.* Internal uncertainty uses 10 000 drug-cluster bootstrap resamples; the LINCS signature-strength threshold (>6 significantly differentially expressed transcripts) was defined without warning probabilities and was not used in warning-score construction. Because it derives from the same expression profiles, it serves as a convergent response-strength label based on metadata defined without using warning probabilities. External analyses use the metadata-selected common condition and drug-level inference; *P*-values are exploratory and unadjusted. The analyses assess warning-score compatibility with strong or stress-associated responses.

After collapsing 305 technical signatures to 128 biological conditions, the common condition (PC3, 24 h, 0.040 μM) was selected from LINCS metadata alone to maximize external-compound overlap before Tox21/ToxCast outcomes were inspected. At this condition, Tox21 any-stress activity had ROC-AUC 0.728 (95% CI 0.573–0.862; 15 compounds). For 12 strictly matched ToxCast compounds, the warning score correlated with continuous cytotoxicity hit fraction (Spearman ρ = 0.727, drug-level bootstrap 95% CI 0.229–0.956; permutation *P* = 0.011); the binary sensitivity analysis gave ROC-AUC 0.719 (95% CI 0.575–1.000; *P* = 0.028). These analyses support compatibility with strong or stress-associated transcriptional responses. A dose-aware cytotoxicity ROC-AUC was not estimable because all 88 overlapping LINCS exposures were below matched cytotoxic concentration lower bounds. In the direct ablation, removing the warning branch reduced global ROC-AUC by 0.009 and increased mean-signature binary NLL by 0.003 in H.9. Taken together, these analyses indicate that the warning score is associated with strong or stress-associated transcriptional responses in the evaluated data and support its use as an empirical abstention signal when target-resolved support is insufficient.

### 4.3 Direct component ablation and same-cell evidence

The target-disjoint component analysis uses the same candidate rows and evaluation design across seven ablation conditions (Tables S16–S17), thereby isolating the contributions of the methodological components. These conditions use 4287 calibration candidate rows and 5512 test candidate rows indexed by 305 chemical signatures, 256 coordinates selected using training targets only, and disjoint 69/16/21 train/calibration/test target identities. The rank-4 near-identity map was estimated only from training targets; its inverse temperature and scalar calibration were selected on the calibration partition, while optimization and conditioning details are provided in Supplementary H.8–H.10. The full model uses target/poly/warning branch masses 0.8/0.1/0.1; a branch-deletion variant omits the corresponding 0.1 working mass without redistribution before joint normalization. All variants use η=0.003 and their own positive-slope binary-logit calibration ($L_{2}=10^{-4}$) fitted only on calibration rows. Absolute performance and paired point estimates are reported in Tables S16-S17. This design is target-disjoint for map fitting and reference-assisted at scoring through fixed evaluation-time target references.

Exact covariance improves mean-signature ROC-AUC over diagonal covariance by +0.011 and over the scale-matched mean-only control by +0.011, with mean-signature binary negative log-likelihood (msNLL) improvements of +0.004 and +0.004. The full learned-alignment/exact-covariance model improves mean-signature ROC-AUC by +0.093 and msNLL by +0.020 over identity/no alignment (Fig. 3a). Proposition 3 implies that the within-signature ROC-AUC contrasts for deleting the warning/null branch, deleting the poly branch, or substituting matched absolute weights are identically zero. For these three contrasts, effects are instead reported as full-minus-ablation global ROC-AUC and ablation-minus-full msNLL: +0.009/+0.003, +0.005/+0.001, and +0.006/+0.002, respectively (Table S17). The separately fitted target-disjoint map has $∥B-I{∥}_{2}=0.950$ and 2-norm condition number $\kappa_{2}(B)=7.100$; these quantities characterize numerical stability, while empirical covariance attribution is provided by the paired contrasts. The paired results separate the component contributions: exact covariance adds a positive effect over the scale-matched mean-only control, the full aligned model yields the larger contrast against identity/no alignment, and the null, poly, and normalization choices act through pooled discrimination, calibration, and branch competition because Proposition 3 fixes their within-signature ranking contrasts at zero.

**Figure 3:**
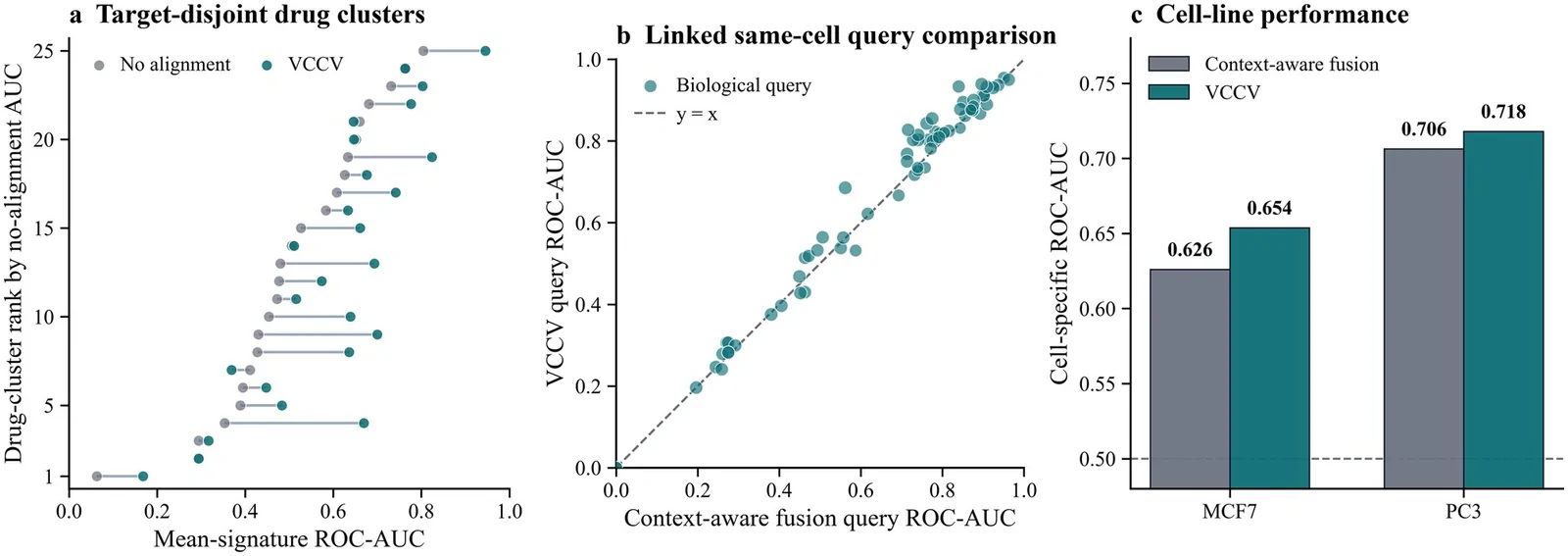
Target-disjoint map-fitting ablation and linked same-cell-supported candidate ranking. Panel a orders drug clusters from the target-disjoint ablation endpoint by identity/no-alignment mean-signature ROC-AUC and shows the corresponding VCCV values; panel b compares per-query ROC-AUC for VCCV and context-aware fusion on the linked endpoint; panel c reports descriptive cell-line ROC-AUC. Linked-endpoint paired effects and confidence intervals are reported in Table S6; component point estimates are reported in Table S17.

The linked same-cell resource carries explicit threshold-defined Davis affinity labels: rows at or above the strong-binding pKd threshold are positive and rows at or below the weak-binding pKd threshold are negative, so no unlabelled or sampled-negative rows enter the endpoint. It contains 91 652 outer-test rows (5023 strong and 86 629 weak), spanning 39 drugs, 95 targets, MCF7, and PC3 (5.481% positive; Supplementary D.4 and Table S11). A biological query is dataset × drug × cell × time × compound dose; technical compound profiles do not define separate queries, and target replicates are collapsed within query. The 75-query outer-test table contains 5739 unique query × target candidates, of which 68 mixed-label queries contribute to within-query metrics. Because the diagnostic pools the union of split-specific top-K sets across 30 outer splits, K = 40 applies within each scoring run rather than to the post-aggregation union. “Same-cell” denotes cell-line concordance. Target anchors are selected by source priority and nearest time only; compound dose in μM is never compared with genetic delivery in μL, ng, or undefined units. Expression uses log2(Level-2 + 1) minus the exact-batch control median on the 785 coordinates shared by GSE70138 and GSE92742, with no zero filling.

On this endpoint (Fig. 3b and c), VCCV reaches ROC-AUC 0.704, versus 0.689 for context-aware fusion and 0.668 for the DTI-score control; gene-rank cosine similarity and weighted target-up/down rank score reach 0.583 and 0.507 on the same rows (Table S8). Across the 68 mixed-label biological queries, the paired VCCV–fusion mean-query ROC-AUC difference is +0.018 (95% CI 0.010–0.027; two-sided sign-flip *P* = 3.0 × 10⁻⁵); drug- and target-cluster estimates are +0.015 (0.005–0.026) and +0.015 (0.006–0.026; Table S6). Mean-query average precision is 0.279 for VCCV, 0.270 for fusion, and 0.171 for DTI score. At five candidates, VCCV improves precision by 0.100 (0.044–0.159), recall by 0.093 (0.038–0.154), and enrichment by 1.423 (0.540–2.366) over DTI score (Table S8A). Ties at the cutoff use the expected hit count under random ordering within the tied set.

### 4.4 Residual ambiguity guides compact follow-up measurement prioritization

The panel stage is evaluated on held-out coordinates using 305 chemical signatures, five prespecified masks, 100 matched random panels per mask, and budgets of 5, 10, 15, 20, and 30 coordinates. Coordinates are hidden before selection; the policy never reads their case values. The full-coordinate posterior calculated within this evaluation agrees with the reference posterior from the target-disjoint analysis to numerical precision in the reported evaluation.

The policy has higher leader recovery than matched-random panels at every budget (Fig. 4a and b; Table S13). Posterior KL, separation, and entropy are treated as secondary diagnostics and are not used for the primary leader-recovery claim. The evaluation tests recovery of a prespecified full-coordinate computational reference under held-out measurements; the policy therefore selects coordinates that preserve the model’s own leading hypothesis under masking, converting residual ambiguity into a small, decision-relevant set of measurements.

**Figure 4:**
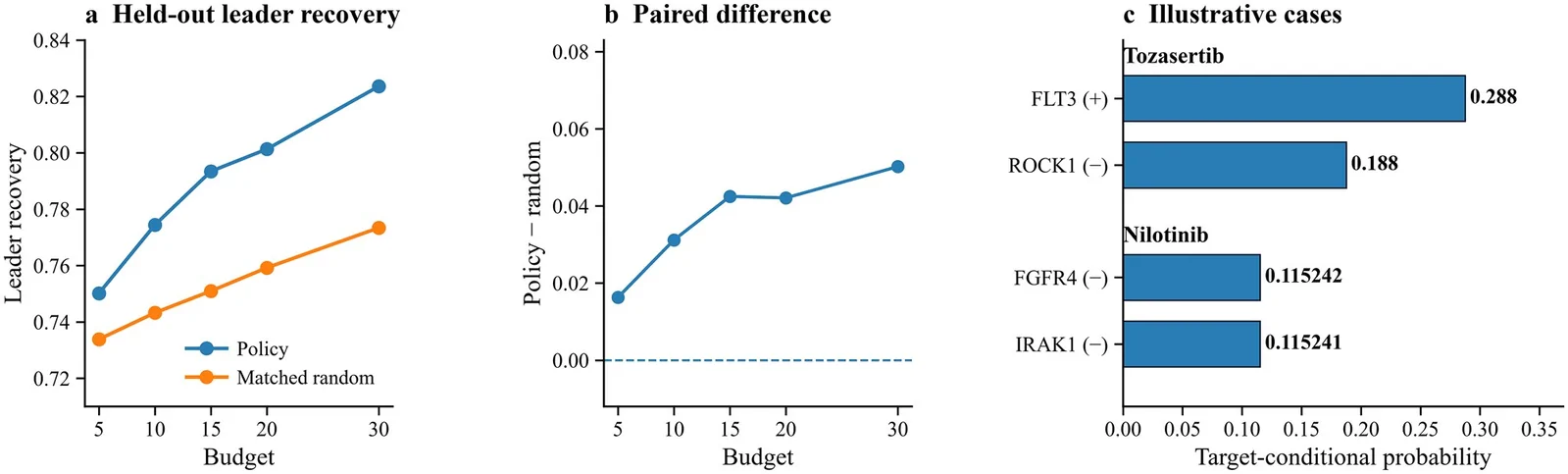
Held-out-coordinate measurement prioritization. Panels show (a) policy and matched-random full-coordinate leader recovery across budgets, (b) paired recovery differences, and (c) full-model target-conditional probabilities for two illustrative cases. The full-coordinate leader is the reference used by the evaluation.

Figure 4c shows two illustrative cases from the target-disjoint analysis. For tozasertib in MCF7 at 3 h and 0.120 μM, FLT3 (label 1) ranks above ROCK1 (label 0), with target-conditional probabilities of 0.288 and 0.188 and a target-conditional margin of 0.100. Under mask M01, a budget-20 panel preserves FLT3 as the leader with a masked margin of 0.081. For nilotinib in MCF7 at 3 h and 0.040 μM, FGFR4 and IRAK1 (both label 0) have probabilities of 0.115242 and 0.115241; the two targets form a near-tie. Table S14 provides the complementary mask-specific details.

## 5 Discussion

VCCV provides a principled corroboration framework for computational target nomination. DTI models generate candidates; context-aligned transcriptomic evidence updates those candidates; a weighted empirical warning mixture prevents forced interpretation under global mismatch; and a follow-up panel converts residual ambiguity into a tractable next experiment. Together, these elements constitute a distinct decision layer that improves retrospective ranking and turns unresolved uncertainty into an actionable measurement strategy. The formal results justify the design choices separately: Theorem 1 concerns exact covariance transport under affine coordinate changes, Corollary 1 quantifies target-mass normalization, and Corollary 2 identifies the limitation of a target-normalized posterior lacking an external rejection channel.

The target-disjoint map-fitting ablation separates the components that this endpoint can attribute. Exact covariance provides an additional positive paired effect over the scale-matched mean-only control, and the full learned-alignment/exact-covariance model has positive paired effects over identity/no alignment on signature-level discrimination and proper scoring. The null, poly, and prior-normalization variants preserve within-query single-target order by Proposition 3, but can change pooled discrimination, calibration, target-versus-null/poly competition, posterior gaps, and returned states; their global ROC-AUC and msNLL effects are reported in Table S17. Together, these results identify complementary roles for covariance-aware alignment and the competing non-target branches.

Current evidence is kinase-centred and reference-assisted. The linked resource covers 39 drugs, 95 targets, MCF7, and PC3; broader target classes and cell lineages remain to be tested. The affine genetics-to-pharmacology map approximates nonlinear and off-target biology, and the warning library is an empirical response model without a toxicity-model interpretation. The internal response-strength analysis has eight positives, and external Tox21/ToxCast overlap is limited. These constraints motivate broader perturbational coverage and prospective target-engagement or rescue assays; within the present retrospective scope, the ranking, calibration, and panel-prioritization results remain supportive.

The three output states imply distinct experimental actions: retain motivates focused validation; abstain suggests revisiting dose, context, or assay modality; and a panel recommendation indicates that a compact discriminative measurement is the preferred next step under the model (Supplementary Section G). For the analysis reported in Section 4.1, the median evaluation time was 30.396 ms per query (IQR 20.760–49.270; 95th percentile 65.461 ms) on one CPU thread, excluding model training and time spent reading or writing data (Supplementary Sections C.2–C.4). After the associated low-rank (Woodbury–Sylvester) solves, the single-target alignment-induced covariance update is evaluated in an augmented latent system of dimension at most eight, so no dense 256 × 256 covariance is inverted per single-target hypothesis.

## Supplementary Material

btag694_Supplementary_Data

## Data Availability

DTI labels originate from the public Davis and KIBA kinase resources and were instantiated through the public DeepDTA benchmark archive. Perturbational transcriptomic data originate from LINCS L1000 and were accessed through GEO series GSE70138 and GSE92742 together with the CLUE/LINCS portal. Source code of VCCV is publicly available at https://github.com/bio-ai-source/VCCV.
