# Supplementary material for: VCCV: conservative transcriptomic corroboration for measurement prioritization of computational drug–target hypotheses
Source: Bioinformatics. 2026 Sep 18;42(10):btag694. doi: 10.1093/bioinformatics/btag694 (PMC13633670; doi:10.1093/bioinformatics/btag694)
Supplement: Supplementary file 1 [file btag694_supplementary_data.pdf]

Supplementary Material for

VCCV: conservative transcriptomic corroboration for measurement prioritization of computational drug–target hypotheses

A. Extended methodological details and assumptions

A.1 Query representation, candidate generation, and split consistency

VCCV is a posterior triage layer applied after a pre-trained computational DTI model. Its raw scores are mapped to calibration-split probabilities by model-specific Platt scaling, and a calibration-only selector is used when multiple score channels are available. The top K=40 targets are retained. Let $w_{\mathrm{h}}(x)>0$ denote a calibrated working weight and $S_K(x)=\sum_{g\in \mathrm{K}(x)} w_g(x)$. The fixed-mass rule is $\pi_{\mathrm{h}}^{\mathrm{fix}}(x)=0.8w_{\mathrm{h}}(x)/S_K(x)$; the optional polypharmacology branch and weighted empirical warning/null branch each receive mass 0.100. The matched H.9 control uses $\pi_{\mathrm{h}}^{\mathrm{abs}}(x)=0.8w_{\mathrm{h}}(x)$ while leaving non-target masses unchanged. Unless weights are normalized over the complete target universe, S_K is a retained-weight sum rather than probability mass. If the optional polypharmacology branch is disabled, its 0.1 working mass is omitted rather than redistributed; the remaining branch weights are normalized only by the joint posterior denominator.

The single-target space contains target-mode pairs. The optional polypharmacology branch moment-matches a same-mode competitive subset of retained targets selected by the prespecified rule below, whereas the warning/null branch averages a training-only prototype library with cluster-frequency weights. Together, these branches allow VCCV to represent competitive multi-target and globally mismatched responses without forcing a single-target explanation. Specifically, include targets with $w_i\geq 0.25\max_j w_j$, retain at most five constituents, and renormalize their preserved target masses within the subset.

Split consistency is enforced for every fitted or calibrated quantity that could absorb paired compound–target information, including alignment parameters, calibration constants, prototype estimates, variance terms, and virtual-regressor parameters. Labeled entity partitions are defined before fitting. Davis and KIBA contain no measured cell, time, or dose; therefore, context-like and condition-like labels are deterministic sensitivity groupings rather than measured biological conditions. Label-independent reference signatures supplied at evaluation are documented separately in H.4.

In the linked diagnostic, observed/virtual references and alignment quantities use only the designated fitting partitions; held-out candidate labels do not enter fitting. In the target-disjoint analysis described in H.9, 69 training targets determine preprocessing, the rank-4 map, covariance, warning library, and covariance scale; 16 disjoint calibration targets determine the evidence temperature and variant-specific scalar calibration; and 21 test targets are scored only after all quantities have been fixed. Fixed calibration/test target signatures serve as evaluation-time anchors. Thus, this analysis is target-disjoint for map fitting but reference-assisted at scoring.

When the calibrated DTI working weights indicate several highly competitive targets, treating them as mutually exclusive can obscure plausible multi-target engagement. The optional polypharmacology branch addresses this case through Gaussian moment matching over the strongest retained single-target candidates. If w_i denotes the normalized working weight of target i, and (μ_i,Σ_i) denotes its aligned Gaussian moment pair, the poly branch is represented by the following first two moments:

$$\mu_{\mathrm{poly}}=\sum_{i\in \mathrm{I}} w_i\,\mu_i.$$

$$\Sigma_{\mathrm{poly}}=\sum_{i\in \mathrm{I}} w_i\left[\Sigma_i+\left(\mu_i-\mu_{\mathrm{poly}}\right)\left(\mu_i-\mu_{\mathrm{poly}}\right)^{\mathrm{T}}\right].$$

This construction preserves the first two moments of the mixture while avoiding a combinatorial search over target sets, allowing the polypharmacology branch to be scored on the same footing as single-target and null branches. The weights are proportional to the preserved retained-target masses within the selected same-mode subset. Moment matching supplies the first two moments needed for branchwise scoring and panel construction; the resulting Gaussian summarizes a competitive multi-target explanation but does not identify a unique target combination.

A.2 Observed perturbation reference set

The observed perturbation reference set uses the LINCS L1000 releases GSE70138 and GSE92742 (Subramanian et al. 2017; Stathias et al. 2020). Each source contains 978 measured landmark coordinates; their intersection contains 785 coordinates, and the 193 non-common coordinates are removed without zero filling. Linked preprocessing uses this common coordinate space. Posterior analyses use a specified 256-coordinate subset; in H.9, those coordinates are selected using training targets only. Compound profiles are restricted to LINCS chemical perturbations, and genetic target signatures are restricted to short hairpin RNA knockdown and gene overexpression perturbations.

Metadata parsing recovers compound or target identity, cell line, perturbation class, exposure time, source, platform, batch, replicate quality, and the native dose field. Compound doses are retained only when recorded in micromolar units. Genetic delivery amounts remain in µL, ng, or undefined units and are never compared with compound concentration. Compound and target identifiers are harmonized to canonical structures and gene symbols before linkage.

For each profile, expression is computed as $\log_2$(Level-2+1) minus the exact-batch matched-control median, followed by source/domain robust scaling and clipping to [-8,8]. Replicate quality is retained as metadata rather than used to re-center positive pairs. The curated resources contain 5,274 real compound profiles and 5,643 genetic target profiles; the mechanism reference collection contains 5,000 drug profiles, 1,377 genetic perturbation profiles, and 711 candidate warning/null profiles before H.9 eligibility filtering. Of these candidates, the 444 training-only profiles satisfying the eligibility rule in A.5 enter the three-prototype warning mixture.

Observed target anchors are aggregated by target, cell context, perturbation mode, source, and exposure time. Native genetic-delivery metadata are retained for provenance but do not participate in cross-modality matching. Heterogeneity among eligible anchors contributes to both the fused mean and the uncertainty estimate.

A.3 Virtual perturbation reference model and variance decomposition

Coverage of the observed reference set alone is insufficient for broad transcriptomic triage. Many target–context–condition combinations are missing, and strict same-cell matching is especially sparse. The virtual reference model addresses this limitation by providing a context-conditioned expected signature together with explicit uncertainty. It summarizes what the existing perturbation resource implies about plausible target-conditioned responses while preserving uncertainty as an explicit component of posterior scoring.

For each candidate h, the virtual reference model provides a gene-wise mean and uncertainty from a split-consistent conditional heteroscedastic regressor using target, cell context, perturbation mode, and exposure time. No cross-modality dose covariate is used. Target, context, and mode embeddings are combined with time and passed through a two-layer ReLU network with separate mean and log-variance heads. Chemical metadata, protein embeddings, and text annotations remain confined to the upstream DTI layer.

Training minimizes gene-wise Gaussian negative log-likelihood (gene-wise Gaussian NLL) with a softplus variance link and a fixed standard-deviation floor σmin=0.02 (equivalent variance floor 4×10^{-4}). A single virtual-variance multiplier is selected on calibration data. Predictions are restricted to the fitted target/context vocabulary; a branch is omitted when neither a supported virtual prediction nor a usable observed anchor exists. Consequently, held-out targets require fixed evaluation-time references and are not treated as reference-free extrapolations.

Observed-anchor selection is deterministic. Candidates must match target, cell line, and perturbation mode; same-source records are preferred, and the nearest standardized exposure time is selected within the source tier. Platform and batch disagreements contribute fixed penalties. Compound concentration never enters this distance.

Anchor quality is $q_a=0.7\,\mathrm{clip}[(\mathrm{distil\_cc\_q75}+1)/2,0,1]+0.3\{1-\exp[-\max(\mathrm{TAS},0)/3]\}$; metadata coverage is 100% for compound and target anchors. The mapping distance is $d_{\mathrm{map}}=1.00I(\text{different source})+0.30\Delta_{\mathrm{time}}+0.40I(\text{different platform})+0.20I(\text{different batch})$, with missing categorical fields treated as mismatches. The observed weight is $\omega_a=\mathrm{sigmoid}\{\mathrm{logit}[10^{-4}+(1-2\times10^{-4})q_a]-d_{\mathrm{map}}\}$; ties are resolved by source tier, time distance, quality, and stable signature identifier.

Observed and virtual moments are fused before alignment, preserving mean mismatch, anchor heterogeneity, and support scarcity as distinct uncertainty sources.

Coordinate-wise moment fusion is $\mu_{\mathrm{fuse}}=\omega\mu_{\mathrm{obs}}+(1-\omega)\mu_{\mathrm{virt}}$ and $v_{\mathrm{fuse}}=\omega\left[v_{\mathrm{obs}}+(\mu_{\mathrm{obs}}-\mu_{\mathrm{fuse}})^2\right]+(1-\omega)\left[v_{\mathrm{virt}}+(\mu_{\mathrm{virt}}-\mu_{\mathrm{fuse}})^2\right]$. The between-source terms preserve dispersion, and the rule reduces to the observed or virtual moments at $\omega=1$ or 0.

Section B.1 derives the full covariance transport used for posterior inference. Because $B-I$ has numerical rank 4, the single-target quadratic form and log determinant are evaluated through a low-dimensional Woodbury reduction whose augmented latent dimension is at most $2r=8$; no dense covariance is inverted per single-target hypothesis. The covariance-simplification control is evaluated in the H.9 ablation.

A.4 Constrained alignment map and estimation objective

The fitted alignment uses one shared map $B=I+UV^{\top}$ on $G=256$ analysis coordinates. Its update has numerical rank 4. Mode $m\in\{\mathrm{LoF},\mathrm{GoF}\}$ enters through a positive scale β_m and translation b_m, so $\mu_{\mathrm{h}}=\beta_m\left(B\mu_{\mathrm{fuse,h}}+b_m\right)$. The shared map acts on the fused perturbational reference, not on the observed query. For the fit used in Section 4.1, $\beta_{\mathrm{LoF}}=0.570$ and $\beta_{\mathrm{GoF}}=0.550$; the separately fitted target-disjoint scales are reported in H.9.

$$\min_{U,V,\beta_m,b_m}\sum_i \|u_i-\beta_{m_i}\left((I+UV^T)x_i+b_{m_i}\right)\|_2^2+\lambda_{reg}\left(\|U\|_F^2+\|V\|_F^2\right),$$

In the alignment objective, the shared low-rank factors U and V are fitted jointly, while scale and bias are mode specific. The Frobenius penalty discourages departure from identity. For the fit used in Section 4.1, $\|B-I\|_2=0.761<1$, so no projection was required; Table S18 reports its diagnostics. The target-disjoint fit is separate, uses a 0.95 spectral bound with 55 projections during estimation, and is reported in H.9-H.10.

$$\widetilde{\Sigma}_{\mathrm{h}}=T_m diag\left(v_{fuse,\mathrm{h}}\right)T_m^{\top}+\Sigma_{base},\qquad \Sigma_{\mathrm{h}}=s\widetilde{\Sigma}_{\mathrm{h}}.$$

For a single-target branch, the fused gene-wise variance v is transported as $\Sigma_{\mathrm{h}}=s\left[\beta_m^2 B\mathrm{diag}(v)B^{\top}+\Sigma_{\mathrm{base}}\right]$. The optional polypharmacology covariance is obtained by the moment-matching rule in A.1 from the corresponding single-target moment pairs, including between-mean dispersion. For the analysis in Section 4.1, the branch covariance scale $s=0.033$ is estimated by Gaussian maximum likelihood from 292 matched single-target residual profiles in the drug-held-out training partition used for that fit; no calibration or test profile enters this estimate. In a training-only half-split assessment, the fit half estimated $s=0.035$ and the untouched validation subset implied 0.0314. Quadratic forms and log determinants are evaluated through the Woodbury reduction in B.1; marginal variances are retained only for compact descriptive output and are not substituted into the likelihood. The calibration-only virtual-variance factor in A.3 is a separate quantity.

In the linked resource, compound signatures are matched to target signatures by same cell, source priority, and nearest exposure time. Compound dose defines the biological query and is excluded from comparison with genetic-delivery metadata. The alignment map is used as an empirical local domain-adaptation device, and all alignment pairs and linked labels are retrospective; no causal transport interpretation is assigned.

A.5 Warning/null branch and calibration protocol

The warning/null branch is a cluster-frequency-weighted empirical mixture. Component energies are combined as $E_{\perp}(u)=-\log\sum_k w_k\exp[-E_{\perp k}(u)]$, so the branch log weight is $\log\pi_{\perp}+\eta\log\sum_k w_k\exp[-E_{\perp k}(u)]$. Post hoc program annotations are descriptive only. A high warning probability supports abstention under global mismatch; it does not diagnose toxicity or exclude biochemical target engagement.

The training warning library contains 444 profiles assigned to three prototypes with counts 156, 177, and 111 and fixed weights $w=(0.351,0.399,0.250)$. Each component uses its training-only prototype and covariance; the log-sum-exp is evaluated before applying the branch-level energy scale η. Calibration/test responses do not enter prototype estimation. Training-only eligibility rule: candidates are training-only LINCS chemical-perturbation profiles with no eligible same-cell target anchor and minimum target-branch fit above the 90th training percentile; compounds and biological conditions used in the 305-signature evaluation are excluded.

Calibration is performed separately for each analysis. For the analysis in Section 4.1, $\eta=0.05$ was determined within its calibration partition and retained with the positive-slope two-stage calibration map (Table S3). For the target-disjoint analysis, $\eta=0.003$ was determined independently within its calibration partition and is used with a positive-slope binary-logit calibration with $L_2=10^{-4}$ fitted separately for each variant on the same 4,287 calibration rows. All calibration-derived quantities are fixed before test scoring.

B. Full proofs for Section 2

B.1 Full covariance transport and low-rank evaluation

Let $T_m=\beta_m B=\beta_m\left(I+UV^{\top}\right)$ and $D_{\mathrm{h}}=\mathrm{diag}(v_{\mathrm{h}})$, where $D_{\mathrm{h}}\succ0$ after the variance floor. Define the unscaled branch covariance

$$\widetilde{\Sigma}_{\mathrm{h}}=T_m D_{\mathrm{h}} T_m^{\top}+\Sigma_{\mathrm{base}},$$

and the scored covariance $\Sigma_{\mathrm{h}}=s\widetilde{\Sigma}_{\mathrm{h}}$ with $s>0$ and $\Sigma_{\mathrm{base}}\succ0$. Expanding the push-forward gives

$$\widetilde{\Sigma}_{\mathrm{h}}=\Sigma_{\mathrm{base}}+\beta_m^2 D_{\mathrm{h}}+\beta_m^2 UV^{\top}D_{\mathrm{h}}+\beta_m^2 D_{\mathrm{h}}VU^{\top}+\beta_m^2 U\left(V^{\top}D_{\mathrm{h}}V\right)U^{\top}.$$

Set

$$Q_h=\Sigma_{\text{base}}+\beta_m^2 D_h, \qquad C_h=\beta_m[U \quad D_h V], \qquad M_h=\begin{bmatrix} V^\top D_h V & I_r \\ I_r & 0 \end{bmatrix}.$$

Direct block multiplication yields

$$C_h M_h C_h^\top=\beta_m^2\left[U\left(V^\top D_h V\right)U^\top+UV^\top D_h+D_h V U^\top\right],$$

so that

$$\tilde{\Sigma}_h=Q_h+C_h M_h C_h^\top.$$

The augmented correction therefore has latent dimension at most $2r$; for the fitted r=4 map, the latent system has dimension at most eight. Although M_h is generally indefinite, it is nonsingular because

$$M_h^{-1}=\begin{bmatrix} 0 & I_r \\ I_r & -V^\top D_h V \end{bmatrix},$$

as verified by multiplication. Positive definiteness follows from the original construction rather than from the sign of M_h: for every nonzero $x\in\mathrm{R}^G$,

$$x^\top\tilde{\Sigma}_h x=\left(T_m^\top x\right)^\top D_h\left(T_m^\top x\right)+x^\top\Sigma_{\text{base}}x=\|D_h^{1/2}T_m^\top x\|_2^2+x^\top\Sigma_{\text{base}}x>0.$$

Hence $Q_h\succ 0$ and $\tilde{\Sigma}_h\succ 0$. Applying the Woodbury identity to $Q_h+C_h M_h C_h^\top$ gives

$$\tilde{\Sigma}_h^{-1}=Q_h^{-1}-Q_h^{-1}C_h\left(M_h^{-1}+C_h^\top Q_h^{-1}C_h\right)^{-1}C_h^\top Q_h^{-1}.$$

For the determinant, factor with the positive-definite square root of Q_h:

$$\tilde{\Sigma}_h=Q_h^{1/2}\left[I_G+Q_h^{-1/2}C_h M_h C_h^\top Q_h^{-1/2}\right]Q_h^{1/2}.$$

Therefore

$$\det\tilde{\Sigma}_h=\det Q_h\,\det\left[I_G+Q_h^{-1/2}C_h M_h C_h^\top Q_h^{-1/2}\right].$$

Using Sylvester's identity $\det(I+AB)=\det(I+BA)$ with $A=Q_h^{-1/2}C_h$ and $B=M_h C_h^\top Q_h^{-1/2}$ yields

$$\det\tilde{\Sigma}_h=\det Q_h\,\det\left[I_{2r}+M_h C_h^\top Q_h^{-1}C_h\right],$$

and thus

$$\log\det\tilde{\Sigma}_h=\log\det Q_h+\log\det\left[I_{2r}+M_h C_h^\top Q_h^{-1}C_h\right].$$

Finally,

$$\Sigma_h^{-1}=s^{-1}\tilde{\Sigma}_h^{-1}, \qquad \log\det\Sigma_h=G\log s+\log\det\tilde{\Sigma}_h.$$

Thus exact scoring requires solves and a log determinant for Q_h, followed by a factorization of only a $2r\times 2r$ latent system; it does not materialize or invert a dense $G\times G$ covariance per hypothesis.

B.2 Proof of Proposition 1

For any branch $\mathrm{h}\in\mathrm{H}$, write

$$p_\eta(\mathrm{h}|u)=\frac{\pi_h\exp[-\eta E_h(u)]}{Z(u)}, \qquad Z(u)=\sum_{g\in\mathrm{H}}\pi_g\exp\left[-\eta E_g(u)\right].$$

For branches h and h' with positive prior mass, division cancels the common normalizer:

$$\frac{p_\eta(\mathrm{h}|u)}{p_\eta(\mathrm{h}'|u)}=\frac{\pi_h}{\pi_{h'}}\exp\{-\eta[E_h(u)-E_{h'}(u)]\}.$$

Taking logarithms proves

$$\log\frac{p_\eta(\mathrm{h}|u)}{p_\eta(\mathrm{h}'|u)}=\log\frac{\pi_h}{\pi_{h'}}-\eta[E_h(u)-E_{h'}(u)].$$

Setting $\mathrm{h}'=\perp$, the requirement that h exceed the warning branch by log-odds margin δ is

$$\log\frac{p_\eta(\mathrm{h}|u)}{p_\eta(\perp|u)}\geq\delta.$$

Substitution and rearrangement give the equivalent energy condition

$$E_\perp(u)-E_h(u)\geq\eta^{-1}\left[\log\frac{\pi_\perp}{\pi_h}+\delta\right].$$

Hence the transcriptomic energy advantage must overcome both prior odds and the requested decision margin.

B.3 Proof of Theorem 1 and Corollaries 1–2

Let $u'=Au+b$, with A invertible, and transform a Gaussian branch by $\mu_h'=A\mu_h+b$ and $\Sigma_h'=A\Sigma_h A^\top$. Then

$$u'-\mu_h'=A\left(u-\mu_h\right), \qquad (\Sigma_h')^{-1}=A^{-\top}\Sigma_h^{-1}A^{-1}.$$

Consequently,

$$\left(u'-\mu_h'\right)^\top(\Sigma_h')^{-1}\left(u'-\mu_h'\right)=\left(u-\mu_h\right)^\top\Sigma_h^{-1}\left(u-\mu_h\right).$$

Moreover,

$$\log\det\Sigma_h'=\log\det\left(A\Sigma_h A^\top\right)=\log\det\Sigma_h+2\log|\det A|.$$

Every Gaussian branch energy therefore changes by the same additive constant $\log|\det A|$. If every warning prototype is transformed by $v_k'=Av_k+b$ and $\Omega_k'=A\Omega_k A^\top$, then each component energy obeys $E_{\perp k}'=E_{\perp k}+c$, with $c=\log/\det A/$. The weighted mixture consequently satisfies

$$-\log\sum_k w_k\exp[-E_{\perp k}']=-\log\sum_k w_k\exp[-(E_{\perp k}+c)]=E_\perp+c.$$

Thus every branch receives the same energy constant, which cancels from all posterior-odds ratios. This proves the forward invariance statement.

For uniqueness, restrict attention to positive-definite covariance-only transforms $T_A(\Sigma)$ that depend only on A and Σ, with the affine mean map, priors, and $\eta>0$ fixed. Assume preservation of all Gaussian energy differences for every positive-definite Σ,Σ_0 and every

transformed-coordinate residual vector r. Comparing two branches with a common mean and subtracting the equality at r=0 isolates the quadratic terms and gives

$$r^{\top}\left[T_A(\Sigma)^{-1}-T_A(\Sigma_0)^{-1}\right]r=r^{\top}A^{-\top}\left(\Sigma^{-1}-\Sigma_0^{-1}\right)A^{-1}r.$$

Because this holds for every r, equality of symmetric quadratic forms implies

$$T_A(\Sigma)^{-1}-T_A(\Sigma_0)^{-1}=A^{-\top}\left(\Sigma^{-1}-\Sigma_0^{-1}\right)A^{-1}.$$

Hence there is a symmetric matrix C_A, independent of Σ, such that

$$T_A(\Sigma)^{-1}=A^{-\top}\Sigma^{-1}A^{-1}+C_A.$$

Writing $P_A=A^{\top}C_AA$ and inverting gives

$$T_A(\Sigma)=A\left(\Sigma^{-1}+P_A\right)^{-1}A^{\top}.$$

Preservation of all Gaussian energy differences also requires determinant differences to agree. Substitution yields

$$\operatorname{logdet}T_A(\Sigma)-\operatorname{logdet}\left(A\Sigma A^{\top}\right)=-\operatorname{logdet}(I_G+\Sigma P_A),$$

which must be independent of Σ. Choosing $\Sigma=tI_G$ for every t>0 gives

$$\det(I_G+tP_A)=\prod_{j=1}^{G}\left(1+t\lambda_j(P_A)\right)$$

constant in t. A nonzero eigenvalue would make this polynomial nonconstant, so every eigenvalue of the symmetric matrix P_A is zero. Therefore P_A=0, then C_A=0, and

$$T_A(\Sigma)=A\Sigma A^{\top}.$$

This proves uniqueness within the stated class of branchwise Gaussian covariance transforms.

For Corollary 1, compare

$$\pi_{\mathrm{h}}^{\mathrm{fix}}=\frac{m_1w_{\mathrm{h}}}{S_K},\qquad \pi_{\mathrm{h}}^{\mathrm{abs}}=m_1w_{\mathrm{h}},$$

while holding every non-target branch weight and all energies fixed. For a retained target h and unchanged non-target branch b,

$$\log\frac{p_\eta^{\mathrm{fix}}(\mathrm{h}|u)}{p_\eta^{\mathrm{fix}}(b|u)}-\log\frac{p_\eta^{\mathrm{abs}}(\mathrm{h}|u)}{p_\eta^{\mathrm{abs}}(b|u)}=\log\frac{\pi_{\mathrm{h}}^{\mathrm{fix}}}{\pi_{\mathrm{h}}^{\mathrm{abs}}}=-\log S_K.$$

The shift is target-favouring exactly when S_K<1, neutral when S_K=1, and target-disfavouring when S_K>1. Rearranging Proposition 1 shows that the minimum required energy advantage changes by $\eta^{-1}\log S_K$. Because $1/S_K$ is common to every retained target, target-to-target odds are unchanged.

For Corollary 2, consider a posterior normalized only over target branches and add a common constant c to every target energy. Then

$$\frac{\pi_{\mathrm{h}}\exp[-\eta(E_{\mathrm{h}}+c)]}{\sum_g\pi_g\exp\left[-\eta\left(E_g+c\right)\right]}=\frac{\pi_{\mathrm{h}}\exp(-\eta E_{\mathrm{h}})}{\sum_g\pi_g\exp\left(-\eta E_g\right)}.$$

Common deterioration is therefore invisible to a target-only posterior. An external absolute-fit or rejection branch is required to convert such deterioration into abstention; VCCV supplies that branch through the weighted warning mixture.

B.4 Proof of Proposition 2

Because $L\succcurlyeq 0$, choose a feature factorization $L=\Phi^{\top}\Phi$ and let ϕ_i denote the feature vector for candidate gene i. By Sylvester's identity,

$$J(S)=\operatorname{logdet}(I+L_S)=\operatorname{logdet}\left(I+\Phi_S\Phi_S^{\top}\right).$$

For $i\notin S$, the matrix determinant lemma gives the marginal gain

$$\Delta_i(S)=J(S\cup\{i\})-J(S)=\log\left[1+\phi_i^{\top}\left(I+\Phi_S\Phi_S^{\top}\right)^{-1}\phi_i\right]\geq 0.$$

Thus J is monotone. If $A\subseteq B$, then

$$I+\Phi_B\Phi_B^{\top}\succcurlyeq I+\Phi_A\Phi_A^{\top}\succ 0,$$

so inverse order for positive-definite matrices gives

$$\left(I+\Phi_B\Phi_B^{\top}\right)^{-1}\preccurlyeq\left(I+\Phi_A\Phi_A^{\top}\right)^{-1}.$$

Therefore $\Delta_i(B)\leq\Delta_i(A)$ for every $i\notin B$, establishing diminishing returns and hence submodularity. Under the cardinality constraint $|S|\leq k$, the standard greedy algorithm consequently attains the $1-1/e$ approximation bound. The additional pairwise-correlation screen and ordered relaxation used during panel selection alter the feasible family and are heuristic; that extra screen is not covered by this guarantee.

B.5 Incremental determinant updates for practical greedy search

Let $A_S=I+L_S\succ 0$, and maintain its Cholesky factor $A_S=R_SR_S^{\top}$. For an unselected candidate i, define

$$z_i^{(S)}=R_S^{-1}a_{Si},\qquad r_i^{(S)}=a_{ii}-\|z_i^{(S)}\|_2^2.$$

The block determinant identity gives

$$\det A_{S\cup\{i\}}=\det A_S\,r_i^{(S)},\qquad J(S\cup\{i\})-J(S)=\log r_i^{(S)}.$$

Positive definiteness guarantees $r_i^{(S)}>0$. Suppose candidate j is selected. Set $\alpha_j=\sqrt{r_j^{(S)}}$. The enlarged Cholesky factor is

$$R_{S\cup\{j\}}=\begin{bmatrix}R_S & 0\\ \left(z_j^{(S)}\right)^{\top} & \alpha_j\end{bmatrix}.$$

For each remaining candidate i, forward substitution adds one coordinate,

$$z_{i,\mathrm{new}}=\frac{a_{ji}-\left(z_j^{(S)}\right)^{\top}z_i^{(S)}}{\alpha_j},$$

and the Schur residual updates as

$$r_i^{(S\cup\{j\})}=r_i^{(S)}-z_{i,\mathrm{new}}^2.$$

At selection step s, each candidate update costs $O(s)$, so all candidate marginal updates over k selections cost $O(Mk^2)$. Cholesky initialization and selected-set bookkeeping are bounded by $O(k^3)$, giving the stated $O(Mk^2+k^3)$ search cost after kernel construction. No explicit inverse is formed.

B.6 Proof of Proposition 3

Fix a biological query (u,x) and retained single-target set $H_1(x)$. Write the unnormalized target weight as

$$q_h(u,x)=\pi_h(x)\exp[-\eta E_h(u,x)].$$

Consider a variant v that keeps the candidate rows and all target energies fixed and either removes only branches outside $H_1(x)$ or multiplies every retained target prior by the same positive query-specific factor $c_v(x)$. Then

$$q_h^{(v)}(u,x)=c_v(x)q_h(u,x) \quad \text{for every } h\in H_1(x),$$

with $c_v(x)=1$ when only a non-target branch is removed. For any retained targets h and g,

$$\frac{p_\eta^{(v)}(h|u,x)}{p_\eta^{(v)}(g|u,x)}=\frac{q_h^{(v)}(u,x)}{q_g^{(v)}(u,x)}=\frac{q_h(u,x)}{q_g(u,x)}=\frac{p_\eta(h|u,x)}{p_\eta(g|u,x)}.$$

The common factor and the joint posterior normalizer both cancel. Thus strict ordering and exact ties among the same single-target rows are preserved. Any statistic depending only on that ordering, including within-query ROC-AUC, average precision, reciprocal rank, precision at a fixed rank, and top-k order, is identical. A strictly increasing scalar calibration applied uniformly within a variant also preserves the order, even when its fitted numerical parameters differ across variants.

The conclusion does not extend to global pooled discrimination, binary-label NLL, calibration, posterior gaps, target-versus-null or target-versus-poly competition, or returned decision states. In H.9, deleting the warning branch, deleting the poly branch, and replacing fixed target-mass normalization with matched absolute weights satisfy the proposition's premises; their within-query ROC-AUC contrasts are therefore zero.

B.7 Proof of Proposition 4

Let Σ_h be the exact positive-definite covariance and $\widetilde{\Sigma}_h$ an approximating positive-definite covariance for the same mean μ_h. The pointwise energy distortion is

$$\delta_h(u)=\frac{1}{2}(u-\mu_h)^\top\left(\widetilde{\Sigma}_h^{-1}-\Sigma_h^{-1}\right)(u-\mu_h)+\frac{1}{2}\log\frac{\det\widetilde{\Sigma}_h}{\det\Sigma_h}.$$

The energy distortion $\delta_h(u)$ can have either sign for a particular query. Under the exact branch model $U|h\sim N(\mu_h,\Sigma_h)$,

$$E\left[(U-\mu_h)^\top A(U-\mu_h)|h\right]=\mathrm{tr}(A\Sigma_h)$$

for every fixed matrix A. Substitution gives

$$E[\delta_h(U)|h]=\frac{1}{2}\left[\mathrm{tr}\left(\widetilde{\Sigma}_h^{-1}\Sigma_h\right)-G+\log\frac{\det\widetilde{\Sigma}_h}{\det\Sigma_h}\right]=D_{KL}\left[N(\mu_h,\Sigma_h)\parallel N(\mu_h,\widetilde{\Sigma}_h)\right]\geq 0.$$

The Gaussian Kullback–Leibler divergence is zero if and only if $\widetilde{\Sigma}_h=\Sigma_h$, proving the equality condition.

For strict marginal diagonalization, let $D_h=\mathrm{diag}(\Sigma_h)$ and $C_h=D_h^{-1/2}\Sigma_h D_h^{-1/2}$. Because $D_h^{-1}\Sigma_h$ has trace G and $\det\Sigma_h=\det D_h\det C_h$,

$$E[\delta_h(U)|h]=-\frac{1}{2}\log\det C_h\geq 0,$$

where the inequality follows from Hadamard's determinant inequality and equality holds only when $C_h=I_G$. The pointwise distortion remains query and branch dependent. A fixed prior shift changes only an additive branch constant, and a single global temperature rescales all energy differences uniformly; neither can generally remove branch-specific quadratic terms unless the inverse-covariance contrasts satisfy a nongeneric common proportionality. Proposition 4 is therefore a model-expected proper-score result and does not imply pointwise or ROC-AUC dominance.

C. End-to-end inference sequence and computational complexity

C.1 Inference sequence

The calculations follow Algorithm 1 in the main text. VCCV constructs the calibrated top-K target set, appends an optional polypharmacology branch and a mandatory weighted warning branch, obtains observed/virtual moments, applies the near-identity map with exact covariance push-forward, scores all branches jointly, and returns retain, abstain, or panel. For unresolved queries, $A_\Delta(x)=\{h\in H: p_\eta(\hat{h}|u)-p_\eta(h|u)\leq\rho_{dec}\}$; deep near-ties use the log-determinant MFS objective, whereas wider ambiguity uses coverage-regularized variance ordering.

C.2 Complexity of single-target scoring

Let H denote the number of single-target hypotheses, let r=4 be the numerical rank of $B-I$, and define $Q_h=\Sigma_{base}+\beta_m^2 D_h$. If C_Q denotes the cost of solving linear systems with Q_h and evaluating $\log\det Q_h$, then full covariance scoring costs $O(C_Q+Gr^2+r^3)$ per hypothesis and $O(Gr+r^2)$ factor storage beyond branch vectors. When Q_h admits linear-time structured solves, this reduces to $O(Gr^2+r^3)$. The mean map costs $O(Gr)$. The outer scale s contributes only a scalar inverse factor and the additive term $G\log s$ to the log determinant, so it does not alter this complexity. No dense $G\times G$ covariance is inverted per hypothesis.

With the coordinate dimension and alignment rank fixed, computation scales approximately linearly with the number of evaluated hypotheses. The analysis in Section 4.1 used G=256, K=40, one CPU thread, 50 queries per repeat, two warm-up rounds, and ten measured repeats on an AMD Ryzen 9 7900X. Median evaluation time was 30.396 ms/query (IQR 20.760–49.270; 95th percentile 65.461 ms), excluding model training and time spent reading or writing data.

C.3 Complexity of greedy Minimum Falsification Set selection

Let M denote the number of candidate genes and k the panel budget. After the separation kernel has been formed, the greedy search maintains a Cholesky factor of the selected principal matrix together with each remaining candidate's Schur residual. Candidate marginals are updated incrementally without forming an inverse or refactorizing each trial subset.

At selection step s, each remaining candidate update costs $O(s)$; summing over at most M candidates and k selections gives $O(Mk^2)$. Cholesky initialization and selected-set bookkeeping are bounded by $O(k^3)$, so the search costs $O(Mk^2+k^3)$ after kernel construction; the full derivation is given in B.5.

C.4 Additional runtime measurements

Additional wall-clock measurements used the same hardware environment and exclusion rules as C.2. The totals below cover complete analysis batches, including their stated split-specific optimization or bootstrap workloads, and should not be interpreted as per-query scoring times.

Measured times were approximately 53 s for transcriptomic data preparation; 361 s for the linked analyses (ten seeds × four analyses, including split-specific optimization); 259 s for the target-disjoint ablation with seven conditions and 10,000 bootstrap draws; and 108 s for the held-out-coordinate panel evaluation. These batch totals complement the per-query scoring benchmark in C.2.

D. Experimental protocol and data curation

D.1 DTI labels and primary perturbation resources

Davis and KIBA continuous affinities are first aggregated by canonical drug–target pair and only then binarized once. Data curation identified 489 duplicate canonical pairs, nine label-conflict pairs, and one identifier collision, all resolved before splitting. Davis contains 12,000 pairs: 1,037 positive (pKd≥7; Kd≤100 nM) and 10,963 negative (pKd≤5; Kd≥10,000 nM). KIBA contains 12,000 pairs: 11,685 positive (score≥12) and 315 negative (score≤10). Intermediate values are excluded. The analyzed tables are defined before model fitting, and canonical-pair identifiers are retained for reproducibility. After canonical aggregation and thresholding, 12,000 eligible pairs per dataset are selected by a deterministic entity-stratified procedure without class rebalancing or outcome-dependent ranking. The very different prevalences, 8.642% and 97.375%, require dataset-specific precision–recall interpretation.

The benchmark files were obtained from the public DeepDTA archive (Davis and KIBA directories); Davis et al. (2011) and Tang et al. (2014) are cited as the primary sources of the underlying measurements.

Perturbational evidence comes from GSE70138 and GSE92742. Each source contributes 978 measured landmark coordinates; analyses use the 785-coordinate intersection without imputation, and posterior scoring uses a fixed 256-coordinate subset. Public metadata include perturbation type, native dose unit, time, cell, source, platform/batch, and replicate indicators.

Public access to the perturbational resource is through the GEO accession records for GSE70138 and GSE92742 together with the CLUE/LINCS portal. The analysis uses the associated perturbation, signature-quality, gene, cell, compound, and landmark-level Level-2 expression files.

Compound profiles are restricted to LINCS chemical perturbations with doses in µM. Genetic target signatures are restricted to short hairpin RNA knockdown and gene overexpression perturbations; their µL/ng/undefined delivery fields remain provenance metadata and are not treated as concentration. The two perturbation modes are mapped to suppression-like and activation-like references.

D.2 Signature preprocessing and quality control

The 785-coordinate intersection and exact-batch control subtraction are label-independent profile operations. Entity/grouping splits are then fixed; source/domain robust-scaling statistics and all fitted reference, alignment, covariance, and calibration quantities are estimated on the designated fitting partitions and applied without refitting to calibration/test profiles. Expression values are clipped to $[-8,8]$, and the 193 non-common coordinates are removed rather than zero-filled. Replicate quality and TAS are retained as support metadata.

Low-confidence replicate groups are excluded from primary reference-set fitting but retained for sensitivity diagnostics. The absolute-value clipping rate is 0.102% for drug profiles and 0.361% for genetic perturbation profiles, indicating that clipping is rare.

The curated resource contains 5,274 compound profiles and 5,643 genetic target profiles. The mechanism dataset used by the warning and controlled analyses contains 5,000 drug, 1,377 genetic perturbation, and 711 candidate warning/null profiles before eligibility filtering. The 444-profile training warning library used by the scored three-prototype mixture is the A.5 subset that satisfies the fixed training-only eligibility rule. Coverage remains incomplete, motivating observed/virtual anchors and explicit support boundaries.

D.3 Split construction and evaluation scenarios

Training, calibration, and test sets are built at the entity level. The entity-holdout evaluation uses three random seeds; a separate ten-repeat entity-block analysis provides an additional stability assessment. Calibration occupies 15% of entities, and the test set occupies 20%; the remainder is used for model fitting or reference estimation, subject to the split-consistency constraints described above.

Four proxy grouping regimes are retained for sensitivity analysis: drug hold-out, target partition, deterministic context-like grouping, and deterministic condition-like shift. Davis and KIBA do not contain measured cell, time, or dose, so the latter two labels are deterministic grouping variables only and are never interpreted as biological context or exposure generalization. Entity hold-outs enforce zero train/test overlap for the named drug or target entity.

The DTI proxy benchmark, linked same-cell candidate-ranking endpoint, real-compound warning-score evaluation, H.9 component ablation, and panel evaluation have distinct retrospective estimands. End-to-end causal target confirmation remains outside the evaluated scope.

D.4 Linked same-cell evaluation resource

The linked resource harmonizes real LINCS chemical-perturbation profiles with LINCS knockdown and overexpression target profiles and Davis affinity labels. Target anchors must match cell line; source is prioritized, and exposure time is matched by nearest distance. Compound µM dose is not compared with genetic delivery. Strong Davis labels are 1, weak labels are 0, and intermediate affinities are excluded.

The outer-test table contains 91,652 labeled rows: 5,023 strong and 86,629 weak, with prevalence 0.0548. These rows span 39 drugs, 95 targets, MCF7, and PC3. Averaging each linked candidate over repeated outer-test appearances yields 15,998 unique candidates. All weak labels are observed threshold-defined negatives; no negative sampling or positive–unlabelled formulation is used.

A biological query is dataset × drug × cell × time × compound dose. Technical compound signatures do not define separate queries; target technical replicates are averaged within query. The outer-test table contains 75 queries and 5,739 unique query×target candidates; 68 queries are mixed-label and contribute to within-query AUC/AP. Because results pool the union of split-specific top-K candidate sets across 30 outer splits, K=40 applies within each scoring run rather than to the aggregated union. The full linked context contains 128 queries (121 mixed-label). Query×target candidates are unique after aggregation.

Fitted linked quantities obey the split rules in H.4. Evaluation-time target signatures are fixed inputs, so the linked diagnostic is reference-assisted and transductive.

D.5 Warning/null branch initialization and calibration

The fitted warning/null branch uses the three-component weighted mixture in A.5. Null-MAP cases with $p\eta(\perp|u)\geq0.95$ are labelled strong-warning only for descriptive summaries; the threshold has no decision effect. The Section 4.1 and H.9 maps were estimated separately and are analysis-specific.

The warning-score evaluation protocol, including the 305-signature population, response-strength label, and external assay overlaps, is specified in E.7 and main-text Section 4.2; H.5 states the interpretation boundary. Chemical-dose matching excludes genetic-delivery quantities.

D.6 Metric definitions and reporting conventions

Metric names encode their aggregation. Pooled binary negative log-likelihood (pNLL) is rowwise candidate-label log loss on the common DTI benchmark rows. Mean-query binary negative log-likelihood (mqNLL) is candidate-label log loss averaged within each biological query and then across queries. For H.9, mean-signature binary NLL (msNLL) averages candidate-label log loss within each chemical signature and then across signatures; mean-signature ROC-AUC averages over mixed-label signature-level candidate sets. Gene-wise Gaussian NLL is the optimization criterion for virtual-reference training.

ROC-AUC, average precision, precision/recall at five, enrichment, and leader recovery are reported with the sampling unit specified for each analysis. Posterior KL is defined in H.6 as a secondary diagnostic rather than used for the primary panel claim. Main-text performance estimates and confidence limits use three decimal places; near-tie probabilities, scientific-notation P values, and numerical tolerances retain the precision needed to avoid false equality. Supplementary tables retain additional digits for small paired effects.

Resampling schemes and exact sample sizes are stated with the corresponding analyses in E.6–E.7 and H.2–H.3.

E. Supplementary empirical diagnostics

E.1 Scenario-level macro performance

Figure 2b reports the scenario-level mean ΔROC-AUC values. Table S1 defines the grouping basis and interpretive scope of the four regimes, separating entity-based evaluations from deterministic sensitivity groupings.

Table S1. Definition and interpretive scope of the grouping regimes summarized in Figure 2b.

Scenario	Grouping basis / scope
Context-like group hold-out	Deterministic context-like label; sensitivity grouping only.
Drug hold-out	Canonical drug identity; entity hold-out.
Target partition	Target identity; entity partition.
Condition-like group shift	Deterministic condition-like label; sensitivity grouping only.

Note. Davis and KIBA contain no measured cell, time, or dose; the context-like and condition-like labels are deterministic sensitivity groupings.

E.2 Variance across random seeds

Table S2 reports the three-seed results on the drug-held-out Davis benchmark.

Table S2. Three-seed performance on the drug-held-out Davis benchmark (mean±SD).

Model	ROC-AUC	Pooled binary NLL
DeepDTAGen	0.690±0.012	0.126±0.005
DTIAM	0.684±0.014	0.130±0.006
EviDTI	0.672±0.018	0.128±0.007
MolTrans	0.682±0.015	0.134±0.008
VCCV + DeepDTAGen	0.750±0.008	0.115±0.004

Note. The VCCV row is VCCV + DeepDTAGen under this three-seed protocol. Its higher mean ROC-AUC and lower observed standard deviation are descriptive; no formal variance test is claimed. DeepDTA is excluded because it was not evaluated under this protocol.

E.3 Evidence-temperature settings and monotonicity

Table S3 records the evidence-temperature grid, selected value η=0.05, and positive fitted calibration slopes for the analysis in Section 4.1.

Table S3. Evidence-temperature grid and fitted calibration slopes for Section 4.1.

Evaluated η values	Selected η	Monotonicity check
0.01, 0.02, 0.05, 0.10, 0.20, 0.50, 1, 2, 4	0.05	Selected null and single–poly slopes: 0.2108 and 0.6907; all fitted slopes over the evaluated grid were positive

E.4 Alignment rank and domain-gap sensitivity

Table S4 reports calibration MSE and the best regularization for each candidate rank.

Table S4. Exploratory standalone alignment-reconstruction sensitivity.

Formulation	Rank	Standalone calibration MSE	$\|\|B-I\|\|F$	Standalone best λreg
Shared low-rank	4	0.120894	0.787273	0.001
Shared low-rank	8	0.118793	0.807796	0.100
Shared low-rank	16	0.120796	0.852558	0.100

E.5 Perturbation-reference sparsity ablation

Table S5 reports MRR and drug-held-out AUC as retained L1000 support decreases.

Table S5. Perturbation-reference sparsity ablation.

Preserved data	MRR	Drug-held-out AUC
100% (full retained L1000)	0.603	0.750
50%	0.512	0.728
20%	0.385	0.695
5%	0.120	0.612

Note. MRR and AUC decline as retained reference support decreases.

E.6 Corrected linked endpoint and dependence-aware uncertainty

Table S6 reports paired VCCV–fusion differences under three dependence structures. The query-level analysis uses 68 mixed-label biological queries, 10,000 paired bootstrap draws, and a 100,000-draw sign-flip test. Drug- and target-cluster sensitivities resample repeated entities; the mixed-query endpoint contains 36 drug units. All intervals exclude zero, indicating a consistent but modest advantage on the corrected linked same-cell endpoint.

Table S6. Dependence-aware uncertainty for the corrected linked same-cell endpoint.

Inference unit	ΔROC-AUC	95% CI	Additional statistic
Mixed biological queries (n=68)	+0.0183	[0.0102, 0.0267]	Sign-flip P=0.000030; 10,000 bootstrap
Drug clusters (36 mixed-query units)	+0.0154	[0.0051, 0.0264]	10,000 paired cluster bootstrap
Target clusters	+0.0150	[0.0056, 0.0260]	10,000 paired cluster bootstrap

Note. Effects are paired differences on identical target-collapsed query candidates. One-way cluster analyses address repeated drugs or targets but do not eliminate every dependence source.

E.7 Real-compound and external warning-score evaluation

The internal warning-score evaluation uses 305 real LINCS chemical-perturbation signatures (39 drugs), all with micromolar doses, and the H.9 exact-covariance score at η=0.003. The warning-independent strong-response label is a LINCS signature-strength count greater than 6 (eight positives); uncertainty uses 10,000 drug-cluster bootstrap resamples. Because the label is derived from the same profiles, this is a convergent response-strength assessment rather than independent validation.

Table S7. Additional warning-score sensitivity analyses.

Population	Endpoint	Estimate
Same 305 signatures	TAS≥0.2 sensitivity	ROC-AUC 0.552 [0.443, 0.660]
ToxCast sensitivity (n=12)	Hit fraction>0.5	ROC-AUC 0.719 [0.575, 1.000]
Expanded parent mapping (n=13)	Continuous hit fraction	Spearman ρ=0.709 [0.316, 0.910]

Note. Main-text Table 2 reports the primary real-LINCS, Tox21, and continuous ToxCast results; Table S7 reports the complementary TAS, binary ToxCast, and expanded parent-mapping analyses. External P-values are exploratory and unadjusted.

All 88 dose-aware ToxCast overlaps were below the matched cytotoxic-concentration lower bounds, so no dose-aware cytotoxicity ROC-AUC was estimable; the expanded parent-mapping sensitivity in Table S7 includes imatinib.

E.8 Linked candidate-ranking baselines and query-level metrics

Tables S8 and S8A use the identical 91,652 outer rows and the target-collapsed query definition in D.4. Query metrics use the 68 mixed-label queries, and the five-candidate metrics use expected hits under random ordering within a tied cutoff set.

Table S8. Candidate-ranking baselines on the corrected linked same-cell endpoint.

Model	ROC-AUC	PR-AUC
DTI-score control	0.668	0.182
Gene-rank cosine similarity	0.583	0.065
Weighted target-up/down rank score	0.507	0.054
Context-aware fusion	0.689	0.186
VCCV	0.704	0.204

Note. All methods use identical outer rows. Gene-rank cosine similarity and weighted target-up/down rank score receive calibration-only sign orientation and logistic z calibration; query metrics collapse target technical replicates.

Table S8A. Target-collapsed query precision–recall and five-candidate metrics on 68 mixed-label biological queries.

Method	Mean query average precision	Precision@5	Recall@5	Enrichment factor@5
VCCV	0.279	0.256	0.198	2.914
Context-aware fusion	0.270	0.250	0.195	2.860
DTI-score control	0.171	0.156	0.106	1.491

Table S8A intervals use 10,000 paired cluster bootstrap resamples under the target-collapsed query definition. VCCV–fusion query AP resamples the 36 contributing drug units; VCCV–DTI budget contrasts preserve all candidates from each sampled drug unit.

F. Interpretation scope

Terms such as "intervention" and "reference model" are operational. VCCV prioritizes target hypotheses for follow-up using pharmacological and genetic perturbation data collected under nonidentical conditions; its outputs do not establish causal target identity or toxicity. The linked endpoint is reference-assisted, and confirmation requires broader same-cell/time-matched data and prospective target-engagement or rescue experiments.

G. Practical interpretation of the three output states

Retain prioritizes the leading non-null branch for focused validation; abstain indicates that the warning branch dominates the represented hypotheses; and panel recommends a compact follow-up measurement for an unresolved case. The corresponding next steps are target-engagement or rescue testing, reassessment of dose, context, or assay modality, and targeted-expression measurement. These are decision-support states, not causal or toxicity determinations.

H. Robustness, scope, and reproducibility details

H.1 DTI model inputs and terminology

Table S9 records the input modality of each evaluated DTI model. EviDTI includes derived or predicted drug geometry, but none of the five methods receives a solved drug–target complex as input. The main text therefore uses the term "computational DTI model" throughout.

Table S9. Input modalities of the evaluated DTI models.

DTI model	Compound input	Target input
DeepDTA	SMILES character sequence	Protein amino-acid sequence
MolTrans	SMILES-derived substructure tokens	Protein-sequence substructure tokens
DTIAM	Compound SMILES/graph representation	Protein sequence
EviDTI	2D topology plus derived/predicted drug geometry	Target sequence
DeepDTAGen	Molecular graph derived from SMILES	Protein sequence

H.2 Paired inference on common rows across DTI models

The paired analysis evaluates all five DTI models on the same 81,185 deduplicated rows, representing 22,707 dataset instances and 1,653 drug clusters across 24 scenario×seed×dataset strata. ROC-AUC and pooled binary NLL differences use 2,000 paired bootstrap resamples, stratified by scenario×seed×dataset, with drug clusters sampled within each stratum and identical sampled rows retained for the paired methods. All five ROC-AUC intervals exclude zero, and all pooled-binary-NLL reduction intervals are positive. Figure 2a visualizes paired pooled-binary-NLL reduction, while Table S10 reports the paired ROC-AUC and pooled binary NLL estimates and intervals.

Table S10. Paired ROC-AUC and pooled-binary-NLL effects of VCCV on common rows.

DTI model	ΔROC-AUC [95% CI]	Pooled binary NLL reduction [95% CI]
DeepDTA	+0.023 [0.015, 0.031]	0.008 [0.004, 0.012]
MolTrans	+0.037 [0.026, 0.048]	0.029 [0.021, 0.037]
DTIAM	+0.027 [0.018, 0.036]	0.014 [0.009, 0.019]
EviDTI	+0.031 [0.020, 0.042]	0.006 [0.002, 0.010]
DeepDTAGen	+0.021 [0.011, 0.031]	0.004 [0.001, 0.007]

Note. Effects are paired on the same 81,185 rows. Drug-cluster resampling is performed within scenario×seed×dataset strata, and each test instance is counted once within its scenario×seed×dataset stratum.

H.3 Corrected linked endpoint, baseline mappings, and dependence-aware inference

Endpoint construction and aggregate/query-level results are reported in D.4, E.6, and Tables S6, S8, S8A, and S11. This section specifies the baseline mappings and comparator design used on those identical outer rows.

Gene-rank cosine similarity. For each candidate, matched compound and loss-of-function target signatures are converted to ordinal gene ranks, centered, divided by their row standard deviations, and scored by cosine similarity. Missing raw scores receive the calibration-split median. A monotone sign is selected on calibration data, followed by split-specific median/SD standardization and a logistic z projection.

Weighted target-up/down rank score. The 50 largest and 50 smallest genes in the target signature define target-up and target-down sets. Compound values are converted to normalized ranks; within each set, weights decrease linearly from 1.0 to 0.2. The raw score is the negative difference between the weighted target-up and target-down means, followed by the same calibration-only sign and logistic z mapping.

Context-aware fusion is a split-specific class-balanced logistic candidate model using the same linked inputs while omitting the warning branch and panel state. It serves as the closest ranking comparator and is methodologically distinct from a one-component VCCV ablation.

The fusion model uses nine numeric features: calibrated DTI logit, compound–target cosine similarity, Spearman-like rank similarity, absolute time difference, same-source, same-platform, same-batch, anchor quality, and DTI-logit×cosine interaction; cell ID is categorical. The design matrix has nine standardized numeric columns, one missingness indicator for each numeric feature, and two cell one-hot columns (20 total). Missing numeric values are imputed with training-partition medians before standardization; the corresponding missingness indicators remain in the design. Training weights equalize each biological query, then each unique target within query, and finally balance classes. Within each outer split, fitting and regularization use only the corresponding training and calibration partitions. Compound-dose differences and all VCCV-derived energy, posterior, warning/poly score, decision, and panel quantities are excluded from the comparator.

Table S11. Cell-line composition and candidate-ranking AUC.

Cell line	Rows	Strong / weak	VCCV ROC-AUC	Context-aware fusion ROC-AUC	DTI-score ROC-AUC
MCF7	27,936	1,206 / 26,730	0.654	0.626	0.607
PC3	63,716	3,817 / 59,899	0.718	0.706	0.703

H.4 Split consistency and leakage control

Label-independent profile operations are completed first. The relevant labeled entity partition is then fixed before estimating source/domain scaling and all downstream fitted quantities. Davis/KIBA context-like and condition-like groupings are deterministic sensitivities, not measured conditions. The assessment based on the calibrated scores supplied by the published DTI models does not establish how their original training data were partitioned. Evaluation-time target references are documented as inputs rather than fitted quantities.

For linked ranking, reference and alignment quantities use only designated fitting partitions. In the target-disjoint analysis described in H.9, 69 training targets fit preprocessing, map, covariance, and warning components; 16 targets determine the evidence temperature and scalar calibration; and 21 test targets remain untouched until final scoring. Test-target signatures are used only as evaluation-time anchors. No test affinity label or compound–target pair enters fitting. This analysis is target-disjoint for map fitting and transductive with respect to reference access.

Table S12. Data-separation and leakage-control summary.

Component	Training/calibration restriction	Test exclusion
DTI score calibration	Fit on calibration entities only	No test labels
Observed/virtual references	Fitted after the labeled split; evaluation-time references documented by endpoint	No test affinity labels or paired test rows in fitting
Alignment map	Fit on training pairs only	No held-out pair identities
Warning-reference prototypes	Training partition only	No test profiles
Decision thresholds and panel regularization	Decision threshold: calibration split; panel constants and rules fixed before evaluation	No test-stage selection or case-adaptive tuning
Linked target-row diagnostic	Rows disjoint; target vocabulary/reference may persist	No held-out labeled rows used for fitting
Target-disjoint map fit (H.9)	69 training targets fit preprocessing/map/covariance/null; 16 disjoint targets determine the evidence temperature and scalar calibration	21 test-target labels/pairs excluded from all fitting; fixed target signatures used only at scoring

H.5 Real-compound and external warning-score scope analyses

Main-text Table 2 and Supplementary E.7 report the warning-score estimates and sensitivity analyses. Their scope is compatibility with strong or stress-associated transcriptional responses, not toxicity diagnosis or target-engagement confirmation; H.9 provides the direct no-warning ablation.

H.6 Held-out-coordinate panel evaluation

The held-out-coordinate evaluation uses the 305 H.9 chemical signatures, five masks, 100 matched random panels per mask, and budgets 5, 10, 15, 20, and 30. Hidden observations are not read during selection, and the full-coordinate posterior reproduces the H.9 reference to numerical precision. Figure 4a-b and Table S13 report the leader-recovery results. Posterior KL, separation, and entropy are secondary diagnostics and are not used for the primary claim. The fixed policy settings are $\varepsilon w=10^{-6}$, $\lambda panel=10^{-6}$, and $\lambda cov=1.0$; λpanel is the log-determinant ridge, λcov weights the normalized coverage-redundancy term, and none is selected from masked outcomes.

For mask m, let $V_m \subseteq [G]$ denote the 128 coordinates visible to the selection policy and let $H_m=[G]\setminus V_m$ denote the 128 held-out coordinates, where $[G]=\{1,\ldots,G\}$ and $G=256$. Let $\mathrm{E}_m \subseteq H_m$ be the eligible held-out set, determined by training-reference variance fixed before evaluation and the query-specific binary coordinate-availability mask. Let $\mathrm{F}_m(x)$ collect all other fixed, non-hidden inputs available at selection: query metadata and calibrated candidate weights, aligned branch moments (including the documented fixed evaluation-time target anchors), training-derived warning, covariance, and correlation statistics, and the fixed eligibility/correlation rules. At budget k, the policy selects

$$S_{m,k}=\mathrm{P}_k\left(u_{V_m};\mathrm{F}_m(x),\mathrm{E}_m\right)\subseteq \mathrm{E}_m \subseteq H_m.$$

Thus the policy reads numerical query values only through u_{V_m}; fixed evaluation-time target signatures and eligibility information may enter $\mathrm{F}_m(x)$, but they are not hidden chemical-query coordinates, and no numerical entry of u_{H_m} is read before selection. The values $u_{S_{m,k}}$ are revealed only after selection, after which the posterior is calculated on the resulting coordinate set. Write $p_A(\mathrm{h})$ for this posterior on coordinate set A, and let $\mathrm{h}_{\mathrm{full}}=\mathrm{argmax}_{\mathrm{h}} p_{[G]}(\mathrm{h})$. Policy recovery is $\mathbf{1}\{\mathrm{argmax}_{\mathrm{h}} p_{V_m \cup S_{m,k}}(\mathrm{h})=\mathrm{h}_{\mathrm{full}}\}$. Matched-random recovery averages the analogous indicator over 100 panels $R_{m,k}^{(b)} \subseteq \mathrm{E}_m$, $b=1,\ldots,100$, drawn under the same eligibility and correlation constraints. Random-minus-policy KL remains $\mathrm{KL}\left(p_{\mathrm{full}} \| p_{\mathrm{random}}\right)-\mathrm{KL}\left(p_{\mathrm{full}} \| p_{\mathrm{policy}}\right)$, so a positive value favors the policy.

Masks M01-M05 use seeds 20261332-20261336 and each partition the 256 coordinates without replacement so that $|V_m|=|H_m|=128$. Within H_m, eligibility requires training-reference variance exceeding 10^{-6} and an available query coordinate, thereby defining E_m. Random panels use the same E_m, budget, 0.95 correlation ceiling, and relaxation rule.

Table S13. Held-out-coordinate leader-recovery evaluation across five masks and 305 chemical signatures.

Budget	Policy recovery	Matched-random recovery	Policy−random difference
5	0.7502	0.7339	0.0163
10	0.7744	0.7432	0.0312
15	0.7934	0.7510	0.0425
20	0.8013	0.7592	0.0421
30	0.8236	0.7734	0.0502

Note. Values are full-coordinate leader-recovery rates.

H.7 Representative target-supported and near-tie cases

Figure 4c and main-text Section 4.4 report the full-model target-conditional probabilities, margins, labels, contexts, and interpretations for the two illustrative cases. Table S14 provides the complementary mask-specific details: the budget-20 M01 panel preserves FLT3 for the higher-margin tozasertib query, whereas no panel-recovery claim is made for the nilotinib near-tie.

Table S14. Held-out-panel details for the illustrative cases in Figure 4c.

Compound / condition	Held-out mask	Panel budget	Masked-panel result
Tozasertib; MCF7; 3 h; 0.120 μM	M01	20	FLT3 recovered; masked margin 0.081
Nilotinib; MCF7; 3 h; 0.040 μM	Not applied	Not applied	No masked-panel recovery claimed

H.8 Principal parameter choices and concise theoretical properties

Table S15 summarizes the principal settings and their selection or estimation sources.

Table S15. Principal VCCV settings and estimation boundaries.

Parameter	Reported specification	Selection / estimation
Candidate and coordinate scale	Top-K candidates; shared landmark space and fixed analysis subset.	Prespecified candidate limit; H.9 coordinates selected using training targets only.
Observed-anchor fusion and matching	Deterministic quality-weighted observed/virtual fusion.	Same-cell matching with source/time priority; no cross-modality dose comparison.
Alignment complexity	Rank-4 near-identity map; analysis-specific scale and bias.	Regularized training fit; separate sensitivity diagnostic in Table S4.
Uncertainty and numerical safeguards	Exact covariance transport; training-estimated covariance scale.	Positive-variance safeguard; virtual-variance calibration as in A.3.
Branch specification	Fixed target/poly/warning masses; moment-matched poly branch and weighted warning mixture.	Branch rules in A.1 and A.5; no redistribution after branch deletion.
Panel policy	Near-tie log-det panels; coverage ordering otherwise.	Fixed budgets, masks, eligibility and correlation rules; numerical constants in H.6.

Proposition H1 (invertibility and conditioning). Let $M=UV^{\top}$ and suppose $\|M\|_2\leq q<1$. Then $I+M$ is invertible and $\kappa_2(I+M)\leq(1+q)/(1-q)$. **Proof.** For every vector x, $\|(I+M)x\|_2\geq(1-q)\|x\|_2$ and $\|(I+M)x\|_2\leq(1+q)\|x\|_2$. Hence the smallest and largest singular values are bounded by $1-q$ and $1+q$, respectively. The spectral projection in Section A.4 enforces the premise without changing the rank of M.

Corollary H1 (diagonal transport is exceptional). Under an invertible affine transformation A, exact transport $A\Sigma A^{\top}$ preserves every Gaussian energy difference up to a branch-independent constant. Replacing $A\Sigma A^{\top}$ by $\operatorname{diag}(A\Sigma A^{\top})$ preserves the same odds only in coordinate-compatible exceptional cases in which the discarded off-diagonal contributions cancel for every compared branch and query. It therefore cannot satisfy the universal invariance property of Theorem 1.

Proposition 4 diagnostic scope. The fitted-map quantities $\|B\text{-}I\|_2$, $\kappa_2(B)$, and rank$(B\text{-}I)$ establish nontriviality, conditioning, and low dimension. Branch-specific covariance attribution is provided by the paired H.9 contrasts in Tables S16-S17.

Matched absolute-weight comparison. Under $\pi_h^{fix}=m_1 w_h/S_K$ and $\pi_h^{abs}=m_1 w_h$, the fixed rule changes every retained target-versus-unchanged-branch log odds by exactly $-\log S_K$. For S_K=0.8,0.6, and 0.4, the target-favoring shifts are 0.223, 0.511, and 0.916 nats, respectively. In these S_K<1 examples, the minimum transcriptomic energy advantage required to attain a fixed odds margin is reduced by $-\eta^{-1}\log S_K$. These values follow directly from the normalization rule and are not estimated from data. When w is not normalized over a full mutually exclusive target universe, S_K is a retained-weight sum rather than a probability mass.

Across the 305 H.9 test signatures, median S_K=0.640, IQR 0.480–0.790, range 0.190–1.310, and 270/305 (88.5%) of signatures had S_K<1. Thus the fixed-mass rule was target-favouring for most, but not all, test signatures.

H.9 Direct empirical component ablation under target-disjoint map fitting

Every H.9 contrast uses the same target-disjoint resource: 69/16/21 train/calibration/test targets with zero overlap, 4,287 calibration candidates, and 5,512 test candidates indexed by 305 chemical signatures; 194 signature-level candidate sets are mixed-label. Global ROC-AUC uses all rows, mean-signature ROC-AUC uses the 194 mixed-label sets, and mean-signature binary NLL is averaged within each signature and then across all 305 signatures.

Expression is $\log_2$(Level-2+1) minus exact-batch control median, robust-scaled using training source/domain statistics, clipped to [-8,8], and restricted to 256 coordinates selected using training targets only. The target-disjoint map has rank 4, 60 epochs, learning rate 0.01, regularization 0.01, spectral bound 0.95, and 55 projections; $\|B\text{-}I\|_2$=0.950, $\|B\|_2$=1.109, $\sigma_{min}(B)$=0.156, $\kappa_2(B)$=7.100, and MSE_{train}=3.573. β_{LoF}=0.434, β_{GoF}=0.550, bias L_2 norms are 5.127 and 0, covariance scale s=0.923, and η=0.003. Each variant fits its own positive-slope binary logit ($L_2=10^{-4}$) on calibration rows only. The full model uses target/poly/warning branch masses 0.800/0.100/0.100; a branch-deletion variant omits the corresponding 0.100 working mass without redistribution before joint normalization. Candidate rows, split, and evaluation units are identical across variants.

Calibration/test targets use fixed observed genetic signatures only at scoring; no virtual extrapolation is made for targets absent from the training vocabulary. Training targets alone determine preprocessing, coordinates, map, covariance, warning reference, and scale. No test-target affinity label, pair, or signature enters fitting or calibration.

Table S16. Absolute performance on the identical 5,512 candidate rows and 305 chemical signatures in the target-disjoint map-fitting test set. Mean-signature ROC-AUC is the unweighted mean over the 194 mixed-label signature-level candidate sets, and mean-signature binary NLL is averaged over all 305 signatures.

Variant	Global ROC-AUC	Mean-signature ROC-AUC	Mean-signature binary NLL
Full model: exact covariance + top-K normalization	0.741	0.804	0.255
Diagonal-covariance ablation + top-K normalization	0.733	0.793	0.259
Scale-matched mean-only alignment (β^2 retained)	0.731	0.793	0.259
Identity/no alignment	0.648	0.711	0.275
No explicit null branch	0.732	0.804	0.258
No poly branch	0.736	0.804	0.256
Matched absolute-weight rule	0.735	0.804	0.258

Table S17. Paired point estimates on the target-disjoint test set. Mean-signature ROC-AUC uses 194 mixed-label signature-level candidate sets; mean-signature binary NLL uses all 305 signatures. Under Proposition 3, the final three within-signature ROC-AUC contrasts must be zero.

Paired contrast	Δ mean-signature ROC-AUC	Δ global ROC-AUC	Mean-signature binary-NLL improvement
Exact covariance vs diagonal covariance	0.0108	0.0085 (descriptive)	0.0036
Exact covariance vs scale-matched mean-only alignment	0.0106	0.0102 (descriptive)	0.0035
Full learned-alignment/exact-covariance model vs identity/no alignment	0.0927	0.0927 (descriptive)	0.0200
Explicit null vs no-null	0.0000 (Proposition 3)	0.0090	0.0031
Poly branch vs no-poly	0.0000 (Proposition 3)	0.0049	0.0011
Fixed target mass vs matched absolute weight	0.0000 (Proposition 3)	0.0062	0.0023

ROC-AUC differences are full model minus ablation; binary-NLL improvements are ablation minus full model. By Proposition 3, the final three variants preserve within-signature single-target order and therefore have zero within-signature ROC-AUC contrasts; they can still affect global ROC-AUC, mean-signature binary NLL, calibration, branch competition, posterior gaps, and decisions. Tables S16–S17 show positive mean-signature ROC-AUC and mean-signature binary-NLL effects for exact covariance and learned alignment.

H.10 Fitted-map admissibility diagnostics

Table S18 reports the dimensions, mode-specific scales, update norm, conditioning, and bias norms of the map used in Section 4.1. The update norm is below 1, so no projection is required. The separately fitted target-disjoint map is reported in H.9 and has a different conditioning profile.

Table S18. Fitted-map admissibility and projection diagnostics.

Mode	G / rank / β_m	$\lVert B-I \rVert_2$	$\lVert \beta_m B \rVert_2$ / $\kappa_2(B)$	$\lVert B \rVert_2$ / bias L2	Near-identity criterion
Loss-of-function	256 / 4 / 0.570	0.761	1.001 / 1.805	1.756 / 1.829	Satisfied; no projection
Gain-of-function	256 / 4 / 0.550	0.761	0.965 / 1.805	1.756 / 0	Satisfied; no projection
Aggregate	One shared B	0.761	N/A / 1.805	1.756 / N/A	Both modes satisfied; no projection

Both Section 4.1 modes satisfy the near-identity criterion without projection. The nonzero rank-4 update and $\kappa_2(B)$ summarize nonidentity and conditioning; empirical covariance attribution is provided by the paired H.9 contrasts.
